# Emotion Regulation in Current and Remitted Depression: A Systematic Review and Meta-Analysis

**DOI:** 10.3389/fpsyg.2018.00756

**Published:** 2018-05-18

**Authors:** Endre Visted, Jon Vøllestad, Morten Birkeland Nielsen, Elisabeth Schanche

**Affiliations:** ^1^Department of Clinical Psychology, University of Bergen, Bergen, Norway; ^2^Division of Psychiatry, Haukeland University Hospital, Bergen, Norway; ^3^Solli District Psychiatric Centre (DPS), Bergen, Norway; ^4^Department of Work Psychology and Physiology, National Institute of Occupational Health, Oslo, Norway; ^5^Department of Psychosocial Science, University of Bergen, Bergen, Norway

**Keywords:** emotion regulation, emotion regulation strategies, emotion regulation abilities, self-report questionnaires, major depressive disorder, recurrent depression, meta-analysis, systematic literature review

## Abstract

**Background:** Major Depressive Disorder (MDD) is a highly prevalent, recurrent, and potentially chronic disorder. Identifying risk factors and underlying mechanisms to inform preventive and therapeutic interventions is therefore imperative. Emotion regulation is a proposed factor in the development and maintenance of MDD. The aim of the present review was to summarize and synthesize research on self-reported emotion regulation strategy use and emotion regulation abilities in adults diagnosed with current and remitted MDD.

**Methods:** Seventy-two eligible studies were retrieved from databases through a systematic literature search. Group differences between individuals with current MDD, remitted MDD, and healthy controls were calculated using meta-analytic procedures. Meta-regression analyses investigated potential moderator effects on emotion regulation difficulties.

**Results:** Results indicated that individuals with current MDD report higher maladaptive emotion regulation strategy use for avoidance (Hedges' *g* = 1.3), rumination (*g* = 2.1), and suppression (*g* = 1.1) compared to healthy controls. Also, they reported lower adaptive emotion regulation strategy use for acceptance (*g* = −1.0), problem solving (*g* = −1.0), and reappraisal (*g* = −0.7). Individuals with current MDD reported limited general emotion regulation abilities, indicated by higher alexithymia (*g* = 1.45), lower emotional awareness (*g* = −0.95), emotional clarity (*g* = −1.50) and emotional tolerance (*g* = −1.89). Similar results were found in individuals with remitted MDD for avoidance (*g* = 1.0), rumination (*g* = 1.1), suppression (*g* = 0.6), and general emotion regulation abilities. However, no difference was found between individuals with remitted MDD and healthy controls for adaptive emotion regulation strategies. Meta-regression analyses suggest that age of illness onset, comorbid anxiety and duration of remission influence emotion regulation.

**Conclusion:** The present review and meta-analysis indicates that individuals with current and remitted MDD have difficulties with emotion regulation compared to individuals who have never been depressed. Although depressive symptoms improve, emotion regulation difficulties may continue, and could be a contributing factor to relapse. Our findings inform future research on emotion regulation and psychotherapeutic interventions.

## Introduction

Major Depressive Disorder (MDD) is characterized by symptoms of sustained depressed mood and anhedonia (American Psychiatric Association, 2013). Epidemiological studies show that MDD is a highly prevalent (Ferrari et al., 2013), recurrent (Mueller et al., 1999), and potentially chronic disorder (Kessing et al., 2004). Further, MDD is associated with reduced functioning, lower quality of life and increased risk of suicide (Wittchen et al., 2000; Kessler and Bromet, 2013). Given the magnitude of this public health issue, it is imperative to identify risk factors and underlying mechanisms to inform preventive and therapeutic interventions (Farb et al., 2014). One potential mechanism in the development and maintenance of depression is difficulties with emotion regulation (Donofry et al., 2016). Individuals prone to depression may use maladaptive emotion regulation strategies, where attempts to manage aversive experiences backfire and actually maintain or increase symptoms. In addition, they may be less aware of emotions, have difficulties understanding them, as well as a limited capacity to tolerate them. Consequently, they have problems recovering from negative emotions, resulting in sustained depressed mood (Joormann and Gotlib, 2010). Studies confirm that individuals with MDD employ more maladaptive emotion regulation strategies and less adaptive emotion regulation strategies (Joormann and Stanton, 2016). In addition, emotion regulation difficulties seem to persist after individuals are remitted from MDD (Ehret et al., 2015; Halvorsen et al., 2015). In sum, emotion regulation could be a factor that may contribute to development of both onset and recurrence of depressive episodes.

To our knowledge, there is no meta-analytic evidence of differences in self-reported emotion regulation including both individuals with a diagnosis of MDD and individuals remitted from MDD. Thus, the body of evidence investigating self-reported emotion regulation in individuals with current or remitted MDD has not been systematically reviewed and synthesized. The main aim of the current study was to address this knowledge gap by conducting a systematic review of published research on self-reported emotion regulation, and to quantify self-reported emotion regulation difficulties in people with current and remitted MDD compared to healthy controls through meta-analytic procedures.

### Defining and operationalizing emotion regulation

How can we best understand and conceptualize the ways in which individuals manage their feelings? Several definitions of emotion regulation exist in the literature (Bloch et al., 2010). The most widely used definition states that emotion regulation is the modulation of which emotions one has, when one has them, and how one experiences or expresses these emotions (Gross, 1998b). Deriving from this definition, Gross has proposed a temporal model that specifies a sequence of processes involved in emotion regulation. The sequence involves situation selection (avoiding or approaching certain situations or people), situation modification (active efforts to do something about the situation to alter its emotional impact), attentional deployment (directing one's attention toward more or less emotionally activating aspects of a situation), cognitive change (modifying cognitive evaluations of the situation) and response modulation (direct attempt to influence the ongoing emotional expression or intensity).This sequence can be seen as comprising two main types of emotion regulation, defined in terms of their time of occurrence: antecedent-focused strategies are regulatory processes carried out before the onset of an emotional reaction, while response-focused strategies are carried out after the emotional reaction has been instigated.

Examples of frequently studied antecedent-focused emotion regulation strategies are avoidance, problem solving, rumination and reappraisal. Avoidance is an emotion regulation strategy used to select situations. It is often associated with anxiety disorders, involving behaviors that make it less likely that one will end up in a situation that will give rise to undesirable emotions (Gross, 1998a). For example, a person with a phobia will likely avoid situations that are associated with the feared object. Problem solving is an emotion regulation strategy involving a direct modification of a situation to alter its emotional impact (Gross, 1998b). When the person is in a situation that may provoke an emotional reaction, efforts can be made to prevent this from happening, i.e., rehearsing a speech to prevent anxiety. Rumination is a strategy of attentional deployment, referring to focusing one's attention toward negative emotions and on the implications of these emotions (Nolen-Hoeksema, 1991). Reappraisal is a form of cognitive change that involves interpretation of a potentially emotion-eliciting situation in a way that changes its emotional impact (Gross and John, 2003). Increasing use of reappraisal is an important aim of Cognitive Behavioral Therapy, in that patients are learning to view *in-vitro* or *in-vivo* situations in a new and less emotionally provocative way in collaboration with a therapist.

Examples of common response-focused emotion regulation strategies are acceptance, self-compassion and suppression. Acceptance involves being experientially open to the reality of the present, involving a conscious and active decision to experience what is without trying to modify the experience (Bishop et al., 2004). Acceptance has been proposed as an active ingredient in the third-wave cognitive behavioral interventions of Mindfulness-Based Cognitive Therapy (Segal et al., 2013) and Acceptance and Commitment Therapy (Hayes et al., 2006). Closely related to acceptance is self-compassion, which can be considered an emotion regulation strategy in which painful or distressing feelings are not avoided, but held in awareness with kindness, understanding and a sense of shared humanity (Neff, 2003). While acceptance and self-compassion involves the person to be open to experiences and emotions, suppression involves inhibiting ongoing emotion-expressive behavior (Gross and John, 2003) or experiential emotional reactions (Hayes et al., 2004).

Comprehensive syntheses of experimental studies indicate that emotion regulation strategies differ in how effective they are in reducing negative emotions. In general, reappraisal is more effective than suppression (Augustine and Hemenover, 2009; Webb et al., 2012), and acceptance of negative emotions is a more effective strategy compared to rumination and suppression (Kohl et al., 2012). Moreover, certain emotion regulation strategies seem to be associated with symptoms of psychopathology (Aldao et al., 2010). On this basis, emotion regulation strategies may be categorized as maladaptive (i.e., avoidance, rumination, suppression) or adaptive (i.e., acceptance, reappraisal, problem solving, and self-compassion). However, this distinction is not clear-cut. A given strategy may be more or less functional depending on the situation. For instance, suppressing distressing feelings after receiving upsetting news just before an important exam may facilitate performance, and can thus be adaptive. Conversely, habitually using reappraisal in a context of an abusive relationship may prevent healthy self-assertion and boundary setting. Therefore, flexibility in selection and implementation of emotion regulation strategies in line with the person's own goals and values may be more important than the use of specific strategies *per se* (Aldao et al., 2015). In this sense, the terms adaptive and maladaptive should be regarded as putative rather than definite.

Emotion regulation strategies are easily operationalized, and seemingly easy to both instruct and assess in experimental settings. However, it has been argued that the view of emotion regulation as having primarily to do with discrete strategies to modulate emotional experience is too narrow (Gratz et al., 2015). Focusing exclusively on the specific actions people carry out to regulate emotions may lead to a simplistic view of a complex construct, and to reduced clinical utility. It may be of interest to also assess wider ranging capacities that can impact the management of emotions in important ways. General emotion regulation abilities have been suggested as complementary processes that likely influence the selection and successful implementation of emotion regulation strategies (Tull and Aldao, 2015). Gratz and Roemer (2004) propose such a framework for general emotion regulation abilities consisting of emotional awareness and clarity, and the capacity to tolerate emotions. In order to apply a specific emotion regulation strategy, the person needs to be aware of the emotion, and to be able to understand what the emotion communicates (Sheppes et al., 2015). Also, being easily overwhelmed by emotions may hinder the use of effective and adaptive emotion regulation strategies, and lead to a more rigid use of maladaptive emotion regulation strategies like avoidance (Tull and Aldao, 2015). The theoretical rationale for the significance of general emotion regulation abilities has been articulated by several authors (e.g., Thompson, 1994; Gratz and Roemer, 2004; Berking and Znoj, 2008; Hofmann and Kashdan, 2010). Empirical studies indicate that general emotion regulation abilities including emotional clarity, awareness and tolerance are negatively associated with development of psychopathology (Saarijärvi et al., 2001) and use of maladaptive emotion regulation strategies (Jeffries et al., 2016). Taken together, there is reason to consider general emotion regulation abilities as a contributing factor to the development and maintenance of depression.

Thus, in order to best capture the picture of emotion regulation difficulties in people with current and remitted MDD, this study will focus both on specific emotion regulation strategies (i.e., maladaptive and adaptive), and the general emotion regulation abilities that facilitate them (i.e., emotional awareness, clarity, and tolerance).

### Emotion regulation and major depressive disorder

Given the marked presence of depressed mood and anhedonia in MDD, one hypothesis is that depressed individuals utilize ineffective emotion regulation strategies, and fail to implement effective ones. As a result, individuals with MDD have difficulties in down-regulating sad mood (Joormann and Vanderlind, 2014). Perhaps the most studied maladaptive emotion regulation strategy for people with MDD is rumination. Self-reported rumination is associated with symptoms of depression (Aldao et al., 2010), and people with current and past MDD seem to utilitize this strategy more than healthy controls (Joormann and Stanton, 2016; Liu and Thompson, 2017). Rumination increases the valence and duration of depressed mood, and makes people vulnerable to onset and relapse of depressive episodes (Nolen-Hoeksema et al., 2008). Rumination has also been shown to affect executive functions such as working memory (Watkins and Brown, 2002; Joormann et al., 2011; Meiran et al., 2011) attention (Donaldson et al., 2007) and cognitive control (Koster et al., 2011). Despite rumination being associated with negative outcomes, currently and remitted MDD individuals report positive beliefs about the usefulness of the strategy (Watkins and Moulds, 2005b). Another maladaptive strategy that has been associated with MDD is suppression (Campbell-Sills et al., 2006). Individual studies indicate that people with MDD suppress both positive (Beblo et al., 2012) and negative emotions (Campbell-Sills et al., 2006) more than healthy controls. Further, self-reported habitual use of suppression is associated with symptoms of depression (Aldao et al., 2010), and with increased rumination (Liverant et al., 2011). Thus, an unwillingness to experience emotional reactions may increase the intensity and duration of the response in both experiential and behavioral domains, in addition to increasing the use of other maladaptive emotion regulation strategies. Finally, avoidance has also been suggested to be a maladaptive emotion regulation strategy associated with depression. Although avoidance and suppression may be related constructs, they differ with respect to Gross' process model, in that avoidance is considered an antecedent-focused emotion regulation strategy (i.e., avoiding specific stimuli or situations to prevent emotional reactions from being elicited), whereas suppression is considered a response-focused emotion regulation strategy taking place after the emotional reaction has started to unfold. Studies show that people with MDD report avoiding more emotional experiences than never-depressed controls (Svaldi et al., 2012; Brockmeyer et al., 2015), and that avoidance of emotional experiences is associated with symptoms of depression (Aldao et al., 2010).

Although maladaptive emotion regulation strategies are most predictive of general psychopathology (Aldao and Nolen-Hoeksema, 2010), there is also empirical evidence that people diagnosed with MDD utilize adaptive emotion regulation strategies to a lesser extent compared to healthy controls. In a former meta-analytic study, self-reported problem solving, and reappraisal was negatively associated with symptoms of depression (Aldao et al., 2010). A recent review showed that currently depressed individuals report less use of reappraisal and acceptance of emotions than healthy controls (Liu and Thompson, 2017). Further, studies show that maladaptive emotion regulation strategies can affect the use of adaptive ones. For example, instructed rumination distorted problem solving in depressed individuals in a study by Watkins and Moulds (2005a). Also, a recent meta-analysis of self-reported emotion regulation strategies showed that maladaptive strategies were in general negatively correlated with adaptive emotion regulation strategies (Naragon-Gainey et al., 2017). Recently, self-compassion has been suggested as an important emotion regulation strategy that is particularly effective when coping with depressed mood (Diedrich et al., 2014). Studies suggest that self-reported self-compassion is negatively associated with symptoms of depression (MacBeth and Gumley, 2012), and that self-compassion may be a protective factor for the development of new episodes of MDD (Ehret et al., 2015).

Given the high use of maladaptive emotion regulation strategies, and the interconnectedness between habitual strategy use and general emotion regulation abilities (Tull and Aldao, 2015), currently depressed individuals could have difficulties in emotional awareness, clarity, and tolerance. Deficiencies in emotional awareness and clarity may lead to difficulties in the identification of a feeling and failure of emotion regulation (Gross, 2015). Prior studies have found that emotional awareness and clarity (i.e., alexithymia) is associated with symptoms of depression (Honkalampi et al., 2000). Compared to healthy controls, currently depressed individuals report having limited emotional awareness (Donges et al., 2005), clarity of negative feelings (Thompson et al., 2015), and more alexithymia (Loas et al., 1998; Nandrino et al., 2012). Finally, limited emotional tolerance, i.e., being easily overwhelmed by emotions, may lead to increased use of maladaptive emotion regulation strategies to avoid or suppress emotions (Naragon-Gainey et al., 2017).

In sum, people with current MDD may report more habitual use of maladaptive and less use of adaptive emotion regulation strategies in comparison to healthy controls. It is also likely that these individuals report more difficulties with emotional awareness, clarity and tolerance compared to healthy controls. However, as is pointed out in the literature, symptoms of depression may lead to higher scores on such questionnaires (Treynor et al., 2003). It is therefore also of interest to investigate whether asymptomatic participants with a history of depression still report difficulties in emotion regulation.

### The role of emotion regulation in relapse of major depressive disorder

Given the high relapse rate after recovery of depression (Mueller et al., 1999), emotion regulation difficulties may be a factor that makes people recovered from depression vulnerable to later recurrent episodes. Studies indicate that emotion regulation among individuals with remitted MDD is somewhat similar to those currently depressed. Compared to healthy controls, people who have recovered from depression report using more maladaptive emotion regulation strategies, including rumination (Aker et al., 2014; Halvorsen et al., 2015), suppression (Watkins and Moulds, 2009), and avoidance (Brockmeyer et al., 2012), although the findings on suppression are somewhat contradictory (Joormann and Stanton, 2016; Liu and Thompson, 2017). Findings on adaptive emotion regulation strategies are more inconsistent. Evidence indicates that there is no difference between individuals remitted from MDD and healthy controls in use of reappraisal (Joormann and Stanton, 2016; Liu and Thompson, 2017) and problem solving (Bates and Lavery, 2003). Regarding acceptance, the findings are incongruent. Compared to healthy controls, individuals remitted from MDD report less acceptance of emotions, but similar acceptance of situations (Liu and Thompson, 2017). In sum, the picture that emerges from previous research is that people report using maladaptive emotion regulation strategies after remission from MDD, but the role of adaptive emotion regulation strategies is more unclear.

Further, higher-order emotion regulation abilities could also be of importance. For example, one study indicated that previously depressed individuals report limited general emotion regulation abilities compared to never-depressed individuals (Ehring et al., 2008). A recent study with individuals with remitted MDD showed that higher general emotion regulation abilities following in-treatment predicted lower depressive symptoms at follow-up (Ebert et al., 2017). Another longitudinal study showed that lower self-reported alexithymia (i.e., higher emotional clarity) was associated with remission from MDD (Saarijärvi et al., 2001). Similarly, a study by Rude and McCarthy (2003) found that individuals remitted from MDD reported lower emotional clarity compared to never-depressed individuals.

Overall, individuals with a history of depression may still have a mode of emotional processing that constitutes an inherent vulnerability to developing new depressive episodes, although depressive symptoms are currently at a subclinical level.

### Characteristics of depression that may contribute to emotion regulation difficulties

Recently, emotion regulation has been proposed as a transdiagnostic factor for the development of psychopathology (Fernandez et al., 2016). Comorbidity of anxiety and personality disorders may exacerbate emotion regulation difficulties in participants with current and remitted MDD. Further, studies suggest that early onset age of MDD is associated with early life stressors (Molnar et al., 2001). Overwhelming experiences of distress in early life may contribute to difficulties in emotion regulation, as this may interrupt the development of adaptive emotion regulation skills (Thompson and Goodman, 2010). Consequently, earlier onset age of MDD may exacerbate emotion regulation difficulties. It could also be that total time spent in depressive states will be a maintaining factor, in that depressogenic schemata are easily activated by sad mood (Teasdale et al., 1995). This in turn will lead to new depressive episodes. In this way, the number of former episodes of MDD and duration of remission could be associated with less effective emotion regulation.

### The present review

To our knowledge, there are three recent comprehensive reviews on self-reported emotion regulation in MDD (Aldao et al., 2010; Joormann and Stanton, 2016; Liu and Thompson, 2017). Aldao et al. (2010) found in a systematic review and meta-analysis that symptoms of depression were positively correlated with self-reported maladaptive emotion regulation strategies and negatively correlated with adaptive emotion regulation strategies, with exception of acceptance. However, the literature search was conducted in 2008 and is thus dated. Also, Aldao et al. (2010) did not focus exclusively on samples diagnosed with depression, taking into consideration a broader range of psychopathology as well as samples defined by self-reported symptoms of depression. Similarly, Joormann and Stanton (2016) also reviewed studies with both clinical and non-clinical samples. More recently, Liu and Thompson (2017) reviewed a wide variety of studies investigating emotion regulation strategies in MDD more narrowly defined. They focused their review on people diagnosed with MDD using validated diagnostic interviews. In addition to self-report measures emotion regulation, they also included a number of different study designs including laboratory and naturalistic assessments. The review gives an important summary of the field of emotion regulation in MDD, and offers valuable directions for future clinical work and research. More specifically, they found that individuals with current and remitted MDD report more rumination and less acceptance in comparison to healthy controls. Further, they report that the current status regarding suppression was unclear, as results from studies were not consistent. However, the reviews of Liu and Thompson (2017) and Joormann and Stanton (2016) seem not to be based on systematic literature searches, as no such procedures were reported. Thus, potentially eligible studies might have been omitted. Further, these reviews did not apply meta-analytic procedures to quantify their results. Quantification of findings may elucidate group differences in self-reported emotion regulation. Moreover, such methods render moderator analyses possible, to investigate whether specific characteristics may have an impact on emotion regulation.

In sum, there are a number of considerations that makes the present systematic review and meta-analysis warranted. First, it is important to properly assess the diagnostic status of the participants in these studies to ensure that the participants are actually clinically depressed. A number of studies use only self-report measures, and vaguely described or incomplete interview procedures to diagnose people. Aldao et al. (2010) found only eight studies reporting standardized diagnostic procedures. Therefore, it is an aim of the present review to include only studies utilizing structured clinical interviews to obtain diagnostic status.

Second, it is an aim to quantify the difference between the clinical groups and healthy controls. Comparing properly diagnosed participants to normative (i.e., non-clinical) participants can elucidate current pathological processes that may contribute to development and maintenance of psychopathology. In so doing, it is important to make sure that the healthy control participants do not fulfill criteria for any current psychiatric diagnoses. It is therefore a prerequisite for inclusion that healthy controls are properly screened.

Third, no prior systematic review and meta-analysis has examined emotion regulation in individuals with remitted MDD. One reason why MDD might be associated with endorsement of maladaptive emotion regulation strategies is that persons with MDD may have distorted views of themselves due to the depressive state. They could therefore be more likely to endorse negative or unflattering characteristics than non-depressed people. It is therefore of interest to investigate whether difficulties with emotion regulation persist in people with prior depressive episodes that are now asymptomatic. An aim of the present review is therefore to include both individuals with current and remitted MDD.

Fourth, no prior reviews have taken into consideration general emotion regulation abilities. As previously noted, a mere focus on emotion regulation strategies may yield a too narrow picture of emotion regulation difficulties. Several authors have recommended incorporating such abilities into the research of emotion regulation, as broader capacities such as emotional awareness, clarity, and tolerance could play important parts in the development and maintenance of depression (Augustine and Hemenover, 2009; Tull and Aldao, 2015). Increased knowledge in this domain could inform our understanding of emotion regulation, as well as clinical interventions in useful ways. We thus aim to examine both emotion regulation strategies and general abilities in the present review.

Lastly, it is of interest whether certain characteristics of depression may be associated with emotion regulation difficulties. A final aim of the present review is to examine whether number of prior episodes of MDD, current comorbid anxiety, onset age of MDD and duration of remission may statistically moderate the degree of emotion regulation difficulties.

In line with prior reviews (Aldao et al., 2010; Joormann and Stanton, 2016; Liu and Thompson, 2017), we expected that individuals with current MDD would report more maladaptive and less adaptive emotion regulation strategies compared to healthy controls. We also expected individuals with current MDD to report more limited general emotion regulation abilities including emotional awareness, clarity and tolerance.

Given the recurrent nature of MDD, and the hypothesis that difficulties in emotion regulation may be a vulnerability factor for both onset and relapse of depression, we expected similar results with individuals with remitted MDD. Thus, emotion regulation deficiencies may be a trait-like tendency that is active after symptoms of depression alleviate.

Finally, we expected that the number of prior episodes of MDD, current comorbid anxiety, early onset age of MDD to be positively associated with emotion regulation difficulties, and duration of remission to be negatively associated with emotion regulation difficulties.

## Methods

Methods followed the principles presented by the Preferred Reporting Items for Systematic Reviews and Meta-Analyses (PRISMA) statement (Liberati et al., 2009).

### Protocol and registration

The methods of the analysis, inclusion and exclusion criteria were specified in advance and documented in the PROSPERO International Prospective Register of Systematic Reviews (CRD42015029905).

### Eligibility criteria

Studies were eligible for inclusion if: (1) participants were adult (>18 years old), currently depressed or had a history of depression, as diagnosed using full versions of structured and validated clinical interviews; (2) study design was cross-sectional case-control design, including at least one group of subjects diagnosed with current or remitted depression, and one non-clinical control group, also screened with clinical interviews to assure that the participants were healthy; (3) reported at least one self-report measure of emotion regulation strategy and/or emotion regulation ability; (4) manuscript published in peer-reviewed journals and written in language mastered by the authors (English, German, Norwegian, Swedish and Danish). With regard to exclusion criteria studies were ineligible for inclusion if: (1) participants had severe somatic disorders (i.e., brain damage/injury, severely handicapped, Parkinson's disease, Huntington's disease, dementia) or developmental disorders; (2) study design were *N* = 1 designs or case reports, or lacked a non-clinical, healthy control group; (3) Manuscript was non-published or not peer-reviewed. Although emotion regulation in MDD may be measured using experimental designs, we chose to focus on self-reported questionnaires in an aim to capture the habitual and trait-like emotion regulation tendencies that individuals with current and remitted MDD have.

### Search strategy

The search string was built in collaboration with the first, second, and fourth author after extensive examinations of relevant literature and previous reviews on the field. Several pilot searches were conducted throughout October to November 2015 to secure inclusion of relevant literature. Finally, a university librarian, an expert on systematic literature searches, was consulted to increase quality of the search string. The first author carried out the literature search on the following online databases: Medline, EmBase, PsychINFO using OVID, and Science Citation Index Expanded (1900-), Social Sciences Citation Index (1900-), Arts & Humanities Citation Index (1975-), and Emerging Sources Citation Index (2015-) at ISI Web of Science.

The search string included any combination of relevant terms in the Title, Abstract and Keyword search fields including: Depress^*^, control^*^, ruminat^*^, accept^*^, mindful^*^, suppress^*^, problem solv^*^, avoid^*^, reapprais^*^, self compass^*^, emotion^*^, affect^*^, mood^*^, feel^*^, regulat^*^, repair^*^, manag^*^, compet^*^, clarity, aware^*^, tolera^*^, conscious^*^, differenti^*^, alexithym^*^.

### Study selection

The literature search was conducted December 9, 2015. The study selection process had three stages. First, all findings on databases were exported to the reference managing software Endnote X8 (Clarivate Analytic, 2014). Second, all duplicates were removed using the same software, and abstracts were screened by the first author. At this stage, all studies that reported measuring self-reported emotion regulation ability and strategies in depressed and healthy control samples were included. Finally, the first and second author obtained all full-text manuscripts to independently assess final inclusion. Disagreements between authors were resolved by consensus.

### Data collection process and items

The first author extracted data from the included studies using a constructed form for the purpose. The constructed form was developed and piloted by extracting data from 10 studies. The data extracted from the studies to be used in the qualitative synthesis were: publication year, country and continent, funding, objectives and aims of the study, recruitment procedures, inclusion and exclusion criteria, age of subjects, percentage female, type of diagnostic interview, type of depression symptom checklist, score of depression symptom checklist. The following potential moderators were collected for the quantitative synthesis: means, standard deviations, and number of participants for each emotion regulation outcome, number of previous depressive episodes, duration of remission from depression, comorbidity (anxiety and personality disorders), and age of illness onset.

Twelve corresponding authors were contacted per email to obtain missing data from 15 studies. Two authors replied with missing data and were included for further analyses. To ensure that no dataset had duplicate data, we reviewed with special scrutiny all studies containing the same author(s).

### Summary measures

All meta-analytic procedures, including calculation of summary measures, synthesis of results, and meta regression were executed in the software Comprehensive Meta Analysis version 3 (Biostat, 2014). Due to the nature of our outcome measure (self-report questionnaires), we computed effect sizes based on difference in means. To summarize the outcome measures, we used Hedges' *g*, using the random-effects model, because we expected that the included studies would apply different questionnaires, and thus the scale of measurement would differ from study to study. Further, we expected that many studies would have small sample sizes. Hedges' *g* is a variation of Cohen's *d* that corrects for biases due to small sample sizes (Hedges and Olkin, 1985).

Some studies were anticipated to involve several questionnaires or subscales designed to measure the same concept (e.g., rumination). In these cases, effect sizes for each measure were calculated first, followed by the calculation of the average Hedges' *g* effect size for each study. To interpret the computed effect sizes we used the guidelines by Cohen (1988): a value of ≤0.2 represents a small effect; a value of 0.5 a medium effect and 0.8 a large effect.

### Synthesis of results

Effect sizes were first pooled into distinct categories representing different emotion regulation strategies, in addition to general and specific emotion regulation abilities. Maladaptive (rumination, suppression, and avoidance) and adaptive (acceptance, problem solving, reappraisal, and self-compassion) emotion regulation questionnaires were pooled into one effect size to obtain the general use of maladaptive and adaptive emotion regulation strategies. The distinction between maladaptive and adaptive emotion regulation strategies is theoretically and empirically well-established in prior reviews (Aldao et al., 2010; Joormann and Stanton, 2016; Liu and Thompson, 2017). Also, a recent factor-analysis suggested this distinction to be statistically acceptable (Naragon-Gainey et al., 2017). Consequently, we chose to pool the adaptive and maladaptive strategies into separate categories to obtain a general estimate of each category. Then, all subcategories of emotion regulation strategies were pooled to obtain effect sizes involving use of specific strategies.

Total scores of questionnaires measuring general emotion regulation abilities (e.g., Gratz and Roemer, 2004) were pooled into one effect size. If all subscales within a questionnaire was reported, but not the total score, all subscales were pooled into one effect size to represent the total score. Regarding the specific emotion regulation abilities of emotional clarity, awareness and tolerance relevant subscales were pooled into different effect sizes. In addition, we pooled all questionnaires measuring alexithymia.

Heterogeneity of studies for each pooled effect size was calculated using the Q_within_ statistic. A significant Q_within_ value rejects the null hypothesis of homogeneity. The *I*^2^ statistic was computed as an indicator of heterogeneity in percentages. The *I*^2^ describes the percentage of total variation across studies that are due to heterogeneity rather than chance.

### Risk of bias across studies

To assess publication bias, we conducted the Egger's regression test and Duval and Tweedie's trim and fill procedure. All publication bias analyses were conducted using the software Comprehensive Meta Analysis version 3 (Biostat, 2014).

### Additional analyses

To assess possible moderators of MDD, we planned several meta-regression analyses, using the Comprehensive Meta-Analysis version 3 software (Biostat, 2014). To investigate whether possible moderators had an effect on effect sizes on emotion regulation variables, we ran different regression analyses by applying the following moderators as independent variables: “prior episodes of MDD,” “current comorbid anxiety,” “age of MDD onset,” and finally duration of both current MDD and remission from MDD.

To summarize study and participant characteristics, we used the software SPSS Statistics version 23.

## Results

### Study selection

**Figure 1:**
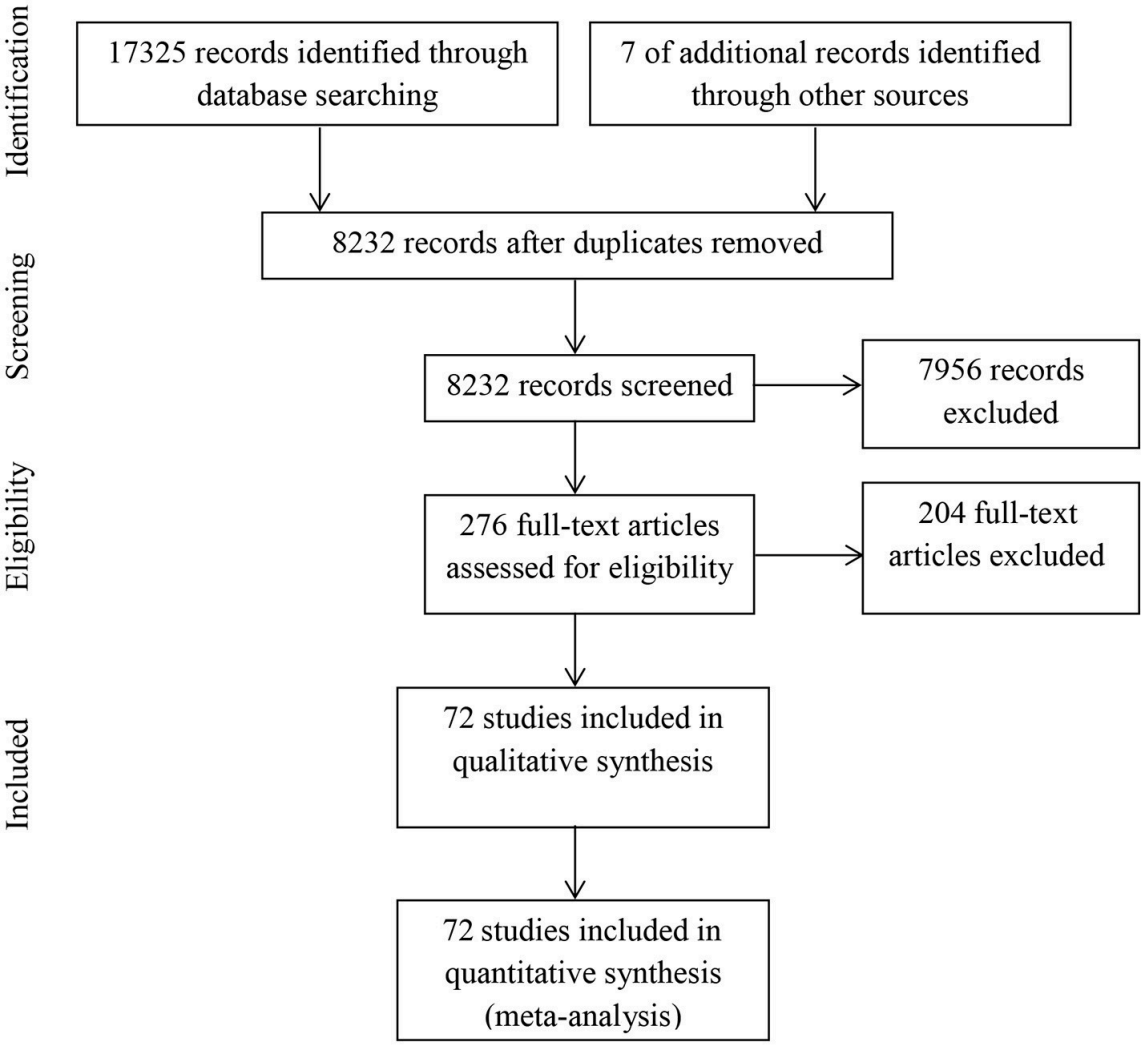
Flow chart of study selection.

A total of 72 studies involving 91 trials were identified for inclusion in our review. A flow-diagram of the study extraction process is provided in Figure 1. Sixty-nine trials involved participants with MDD, and 22 trials involved participants recovered or remitted from MDD. The search in OVID and ISI Web of Science databases provided 17,325 records. After duplicates were removed, 8,225 records remained. Of these, 7,956 records were excluded after reviewing them according to inclusion and exclusion criteria. In addition, seven potentially eligible records were included from reference lists from former studies and reviews. As result, 276 full-text articles were reviewed in more detail by the first and second author. Of these, 59 articles (21%) were rated differently by the authors, and were discussed to reach agreement (in favor of the first author: 32; in favor of the second author: 27). As a result of the rating process, 204 studies were excluded with following reasons: language not mastered by the authors (*n* = 13), clinical group no MDD or history of MDD (*n* = 8), heterogeneous clinical population (*n* = 20), participant age under 18 years (*n* = 9), clinical or healthy control group not screened with diagnostic interview, or not reported applying such an interview (*n* = 95), participants screened partially with diagnostic interview involving only a few diagnostic modules (*n* = 12), control group not healthy (*n* = 5), no self-report measure of emotion regulation (*n* = 17), questionnaire not validated (*n* = 3), duplicate data (*n* = 5), experimental induction prior to assessment (*n* = 3), and corresponding author not returning data on enquiry (*n* = 14). References of all included studies can be found in Appendix A (Supplementary Material).

### Study characteristics

All included studies were published in the English language. The first relevant studies to be published on the topic of this meta-analysis were from 2002 (Watkins and Baracaia, 2002; Watkins and Brown, 2002). Most studies were carried out in the USA (*n* = 23), followed by Germany (*n* = 15), UK (*n* = 10), Canada (*n* = 4), Italy (*n* = 4), Norway (*n* = 3), Australia (*n* = 2), Turkey (*n* = 2), Japan (*n* = 2), and individual studies from Denmark, Iran, Israel, Mexico, South Korea, Sweden, and Taiwan.

### Participants

#### Participants with MDD

In the 69 trials with participants diagnosed with current MDD, an aggregated total of 2,415 participants and 3,536 healthy controls participated. The mean age of the MDD participants was 40.70 years (*SD* = 8.79; range = 20.70–74.00), and mean percentage of women with current MDD was 66.93 percent (*SD* = 14.30%; range = 25–100%). The mean age of the participants in the healthy control groups were 38.53 years (*SD* = 7.22, range = 20.15–69.20), and the mean percentage of women was 64.74 percent (*SD* = 15.8%; range = 32–100%). The majority of studies with MDD patients screened for Axis I diagnoses using the Structured Clinical Interview for DSM Axis I Disorders (SCID I; *n* = 56; First et al., 2002), followed by M.I.N.I. International Neuropsychiatric Interview (MINI; *n* = 6; Sheehan et al., 1998), both MINI and SCID I (*n* = 2), the Schedules for Clinical Assessment in Neuropsychiatry (SCAN; *n* = 2; World Health Organization, 1998) and individual studies using the Diagnostic Interview for Mental Disorders (DIMD; Margraf and Schneider, 2006) and the Primary Care Evaluation of Mental Disorders (PRIME-MD; Spitzer et al., 1994). Depression symptom checklists used were mainly Beck Depression Index (BDI-II; *N* = 41; Beck et al., 1996), followed by the Center for Epidemiologic Studies Depression Scale (CES-D; *N* = 2; Radloff, 1977), the Patient Health Questionnaire (PHQ; *N* = 2; Kroenke and Spitzer, 2002), and individual studies using the Internal State Scale (ISS; Bauer et al., 1991) and the Inventory of Depressive Symptomatology (IDS; Rush et al., 1986). The mean self-reported depression symptom score using the BDI-II was 27.21 (*SD* = 4.32; range = 7.2–34.76). In terms of clinical features of the participants, 15 studies reported a mean duration of current episode of MDD of 26.6 months (*SD* = 23.7; range = 1.6–91.6) and 16 studies reported mean number of previous episodes of MDD (M = 5.29; *SD* = 1.62; range = 2.33–8.10). Eleven studies reported a mean onset age of MDD of 26.92 years (*SD* = 6.17; range = 16.22–36.80). In terms of comorbidity, 37 studies reported percentage of participants with a comorbid anxiety disorder (*M* = 31.40%; *SD* = 27.1%, range = 0–100%). Only one study reported comorbid personality disorders (Riso et al., 2003).

#### Participants with remitted MDD

In the 22 trials with participants with remitted MDD, an aggregated total of 956 individuals with remitted MDD (*M* = 59.68; *SD* = 31.83; range = 15–109) and 1,558 healthy controls (*M* = 296.20; *SD* = 285.77; range = 16–638) participated. The mean age of the participants in the clinical group was 37.50 (*SD* = 5.05, range = 23.60–56.00), and mean percentage of women in the trials were 74.7 percent (*SD* = 12.0%; range = 57.6–100). The mean age of the participants in the healthy control groups were 34.10 (*SD* = 5.21, range = 27.90–48.00), and the mean percentage of women was 61.8 (*SD* = 11.6%; range = 46.9–100). All participants were screened using SCID I (*n* = 21) or MINI (*n* = 1). The majority of trials used BDI-II as a self-report index of depressive symptoms (*n* = 15), and individual studies used CES-D, PHQ, and the Quick Inventory of Depressive Symptomatology-Self Report (QIDS-SR; Rush et al., 2003). The mean self-reported depression symptom score using the BDI-II was 8.37 (*SD* = 2.45; range = 2.60–12.50). In terms of clinical features of the remitted MDD participants, six studies reported a mean duration of remission in months (*M* = 86.24 months; *SD* = 69.85; range = 20.28–176.80), and 14 studies reported a mean number of previous depressive episodes (*M* = 3.77; *SD* = 1.31; range = 1.47–7.30). Eight studies reported depression onset age (*M* = 26.48; *SD* = 5.59; range = 20.39–42.60). With respect to comorbidity, nine studies reported percentage of participants with a current comorbid anxiety disorder (*M* = 13.6%; *SD* = 9.1%; range = 0.0–26.0%) and three studies reported comorbid personality disorders (*M* = 2.2%; *SD* = 6.1%; range = 0.0–19.0%).

### Outcome measures

#### Participants with MDD

Overview of all included studies with trials involving patients with current MDD are presented in Table B.1 in Appendix B (Supplementary Material).

##### Maladaptive strategies

A total of 53 studies reported data from maladaptive emotion regulation strategies from currently depressed participants.

##### Rumination

Thirty-six studies reported data from a total of eight different subscales or questionnaires measuring rumination. The majority of studies applied the Ruminative Response Scale from the Response Styles Questionnaire (RRS; *N* = 34; Nolen-Hoeksema and Morrow, 1991; Treynor et al., 2003). Two studies reported data from the Rumination on Sadness Scale (RSS; Conway et al., 2000), and one study reported data from the subscale Rumination from Leahy Emotional Schema Scale (LESS-RUMINATION; Leahy, 2002). One study reported data from eight questionnaires and subscales measuring rumination, including the RRS and the RSS (Mandell et al., 2014).

##### Avoidance

Sixteen studies reported data from a total of nine different subscales or questionnaires measuring avoidance. Eight studies reported in total 10 trials using the subscale Harm Avoidance from the Temperament and Character Inventory (TCI-HA; Cloninger et al., 1993), followed by one study reporting two trials involving the Avoidance subscale of the Need for Affect Scale (NAS-AVOIDANCE; Maio and Esses, 2001), one study reporting two trials using Avoidance subscale of the COPE (COPE-AVOIDANCE; Carver et al., 1989), one study reporting two trials using Avoidance subscale of the Social Problem-Solving Inventory (SPSI-AVOIDANCE; D'Zurilla and Nezu, 1990) and two studies reporting three trials using the Cognitive Behavioral Avoidance Scale (CBAS; Ottenbreit and Dobson, 2004). Individual studies reported data from the Avoidance subscale from the Impact of Event Scale-Revised (IES-R-AVOIDANCE; Creamer et al., 2003), the Harm Avoidance subscale of the Tridimensional Personality Questionnaire (TPQ-HA; Otter et al., 1995), the Avoidance and Action Questionnaire (AAQ; Hayes et al., 2004), and the Avoidance subscale of Coping Inventory for Stressful Situations (CISS-AVOIDANCE; Endler and Parker, 1994).

##### Suppression

Six studies reported data from a total of 4 different questionnaires or subscales measuring suppression. The majority of studies reported data from the subscale suppression from the Emotion Regulation Questionnaire (ERQ-SUPPRESSION; Gross and John, 2003). Individual studies reported data from the White Bear Suppression Inventory (WBSI; Wegner and Zanakos, 1994), the subscales suppressing negative and positive emotions from the Emotion Acceptance Questionnaire (EAQ; Beblo et al., 2011), and the subscale Suppression from the Inventory of Cognitive Affect Regulation Strategies (ICARUS-SUPPRESSION; Kamholz et al., 2006).

##### Adaptive strategies

A total of 20 studies reported data from adaptive emotion regulation strategies from participants with current MDD.

##### Acceptance

Six studies reported data from six different questionnaires measuring acceptance. Three studies reported data from the subscale Non-accept from the questionnaire Difficulties in Emotion Regulation Scale (DERS-NONACCEPT; Gratz and Roemer, 2004). One study reported data from two trials using the subscale Acceptance of the questionnaire COPE (COPE-ACCEPT), and individual studies reported data using the acceptance subscale of LESS (LESS-ACCEPT), the acceptance subscale of the Cognitive Emotion Regulation Questionnaire (CERQ-ACCEPT; Garnefski and Kraaij, 2007), the acceptance of negative and positive emotions subscales of the EAQ, and the subscales Accept Feelings, Accept Situation, and Mindful Orientation of the ICARUS.

##### Problem solving

Six studies involving eight trials reported data from five different questionnaires measuring problem solving. One study reporting data from three trials reported the total score of the SPSI, and one study reported data from two trials including the subscales Problem Orientation and Problem Solving from the SPSI. Two studies reported data from the Problem Focused and Problem Solving subscales of the COPE (Thompson et al., 2010). Individual studies reported data from the subscale “Task Oriented” from the questionnaire CISS, the Planning subscale from the CERQ, and the Active Coping and Planning subscales of the Brief COPE (Carver, 1997).

##### Reappraisal

Ten studies reported data from five questionnaires measuring reappraisal. Half of the studies (*n* = 5) used the Reappraisal subscale of Emotion Regulation Questionnaire (ERQ-REAPPRAISAL; Gross and John, 2003). Three studies applied the Reappraisal subscale from the Thought Control Questionnaire (TCQ-REAPPRAISAL; Wells and Davies, 1994). Individual studies applied the Cognitive restructuring subscale from COPE, and one study used the subscales Positive reappraisal, Positive Perspective and Positive Refocus from the CERQ and the subscale Positive Reframing from the Brief COPE.

##### Self-compassion

One study reported data from one questionnaire measuring self-compassion. Ehret et al. (2015) utilized the subscale Reassurance from the questionnaire Forms of Self-Criticizing/Attacking and Self-Reassuring Scale (FSCRS-REASSURE; Gilbert et al., 2004).

##### Emotion regulation ability

Four studies measured participants' general emotion regulation ability. Three studies used the DERS, and one study used the Emotion Regulation Skills Questionnaire (ERSQ; Berking and Znoj, 2008). Three studies assessed participants' degree of alexithymia, all using the questionnaire Toronto Alexithymia Scale (TAS-20; Bagby et al., 1994). Finally, two studies assessed participant emotional awareness, clarity and tolerance, using subscales from the DERS.

### Participants remitted from MDD

Overview of all included studies with trials involving patients with a history of MDD are presented in Table B.2 in Appendix B (Supplementary Material).

#### Maladaptive emotion regulation

A total of 21 studies reported data from maladaptive emotion regulation strategies from participants with a history of MDD.

#### Rumination

Fifteen studies reported data from three different questionnaires measuring rumination. The majority of studies reported data from the RRS (*n* = 13). Individual studies used the rumination subscale from CERQ, and the RSS.

#### Avoidance

Six studies reported data from three different questionnaires measuring avoidance. Four studies reported data from the TCI-HA. Individual studies reported data from the NAS-AVOIDANCE and the TPQ-HA.

#### Suppression

Three studies reported data from two questionnaires measuring suppression. Two studies reported data from the ERQ-SUPPRESSION, and one study reported data from the WBSI.

#### Adaptive emotion regulation strategies

A total of seven studies reported data from questionnaires measuring adaptive emotion regulation strategies with participants remitted or recovered from MDD.

#### Acceptance

Two studies reported data from two different questionnaires measuring acceptance. One study used the Acceptance and Action Questionnaire-Revised (AAQ-R; Bond et al., 2011), and one study used the CERQ-ACCEPT.

#### Problem solving

One study measured problem solving, namely the Planning subscale of the CERQ.

#### Reappraisal

Five studies reported data from three different questionnaires measuring reappraisal. Two studies reported data from the ERQ-REAPPRAISAL, and two studies reported data from the TCQ-REAPPRAISAL. Finally, one study reported data from the subscales Reappraisal, Positive Refocus & Perspective from the CERQ.

#### Self-compassion

One study reported data from the subscale Reassurance from the FSCRS-REASSURE (Ehret et al., 2015). Due to the fact that only one study was found in both current and remitted MDD, self-compassion was excluded from further meta-analytic procedures.

#### Emotion regulation ability

Two studies measured participants' general emotion regulation ability, where one applied the DERS and the other the ERSQ. None of the included studies assessed specific emotion regulation abilities of alexithymia, emotional awareness, clarity, or tolerance.

### Synthesis of results

The results including effect sizes, confidence intervals and tests of statistical significance, heterogeneity, and publication bias are presented in Table 1 for current MDD and Table 2 for remitted MDD.

**Table 1:** Synthesis of results from studies with participants diagnosed with current MDD.

		**Pooled** ***n***		**Hedges'**						**Eggers**
	***k***	**MDD**	**HC**	**g**	**95 % CI**	***Z***	***p***	**Heterogeneity**		**intercept**
**ER strategies**								***Q* (betw)**	***I*^2^**	
Maladaptive	54	2,008	2,656	1.771	1.57, 1.97	17.79	<0.001	385.78^***^	86.26	3.06^***^
Avoidance	16	750	1,326	1.288	0.94, 1.64	7.19	<0.001	158.22^***^	90.52	1.97
Rumination	34	1,140	1,171	2.096	1.90, 2.30	20.68	<0.001	128.72^***^	72.81	3.06^***^
Suppression	6	189	251	1.115	0.64, 1.59	4.60	<0.001	24.94^***^	79.96	−6.49
Adaptive	17	724	791	−0.944	−1.20, −0.69	−7.18	<0.001	83.08^***^	80.74	1.52
Acceptance	6	373	399	−1.016	−1.77, −0.26	−2.64	0.008	103.53^***^	95.17	−1.20
Problem solving	6	223	217	−1.036	−1.33, −0.75	−7.00	<0.001	9.01	44.51	−0.73
Reappraisal	10	390	453	−0.701	−0.98, −0.42	−4.89	<0.001	33.69^***^	73.29	1.04
**ER ability**										
General ability	4	135	195	−2.037	−2.69, −1,38	−6.08	<0.001	16.69^**^	82.03	−1.47
Alexithymia	3	78	214	1.451	0.95, 1.95	5.71	<0.001	5.38	62.80	−4.08
Awareness	2	57	102	−0.948	−1.29, −0.61	−5.46	<0.001	0.25	0.00	–
Clarity	2	57	102	−1.495	−1.86, −1.13	−8.07	<0.001	0.04	0.00	–
Tolerance	2	57	102	−1.886	−2.27, −1.50	−9.53	<0.001	0.01	0.00	–

*MDD, Major Depressive Disorder; HC, Healthy Control*.

** p < 0.01

*** *p < 0.001*.

**Table 2:** Synthesis of results from studies with participants in remission from MDD.

		**Pooled** ***n***		**Hedges'**						**Eggers**
	***k***	**rMDD**	**HC**	**g**	**95 % CI**	***Z***	***p***	**Heterogeneity**		**intercept**
**ER strategies**								***Q* (betw)**	***I*^2^**	
Maladaptive	21	849	920	0.996	0.82, 1.18	10.91	<0.001	58.55^***^	65.84	0.66
Avoidance	6	238	353	0.952	0.49, 1.42	4.00	<0.001	30.94^***^	83.84	1.61
Rumination	15	611	567	1.096	0.90, 1.30	10.70	<0.001	34.67^**^	59.62	−3.00
Suppression	3	192	145	0.620	0.15, 1.09	2.56	0.01	8.28^*^	75.85	5.10
Adaptive	6	332	250	−0.202	−0.58, 0.18	−1.04	0.30	24.14^***^	79.28	−3.29
Acceptance	2	59	55	−0.382	−1.43, 0.67	−0.72	0.47	6.34^*^	84.24	–
Problem solving	1	43	39	−0.243	−0.67, 0.19	−1.11	0.27	0.00	0.00	–
Reappraisal	5	316	234	−0.138	−0.55, 0.27	−0.66	0.51	22.16^***^	81.95	−1.23
**ER ability**										
General ability	2	137	668	−0.492	−0.68, −0.30	−5.05	<0.001	0.14	0.00	–

*rMDD, Remitted Major Depressive Disorder; HC, Healthy Control*.

* *p < 0.05*;

** *p < 0.01*;

*** *p < 0.001*.

### Participants with MDD

#### Maladaptive emotion regulation strategies

The pooled effect size (Hedges' *g*) for all maladaptive emotion regulation strategies across all studies involving participants diagnosed with MDD was 1.77 (*p* < 0.001, 95% CI [1.57, 1.97]). This large effect size indicates that participants with MDD report using maladaptive emotion regulation strategies more often than healthy controls. More specifically, all maladaptive emotion regulation strategies were in the large range, including rumination (Hedges' *g* = 2.10, *p* < 0.001, 95% CI [1.90, 2.30]), avoidance (Hedges' *g* = 1.29, *p* < 0.001, 95% CI [0.93, 1.65]) and suppression (Hedges' *g* = 1.12, *p* < 0.001, 95% CI [0.64, 1.59]).

#### Adaptive emotion regulation strategies

The pooled effect size (Hedges' *g*) for all adaptive emotion regulation strategies across all studies involving participants diagnosed with MDD was −0.94 (*p* < 0.001, 95% CI [−1.20, −0.69]). Thus, in general, participants with MDD report using less of adaptive emotion regulation strategies compared to healthy controls. More specifically, effect sizes were large in terms of the adaptive emotion regulation strategies of acceptance (Hedges' *g* = −1.02, *p* < 0.001, 95% CI [−1.79, −0.25]) and problem solving (Hedges' *g* = −1.01, *p* < 0.001, 95% CI [−1.28, −0.74]). The effect size of reappraisal were moderate (Hedges' *g* = −0.70, *p* < 0.001, 95% CI [−0.98, −0.42]).

#### General emotion regulation ability

Although only a minority of included studies did measure participants' general emotion regulation ability, the results indicate that participants with MDD had lower emotion regulation ability in comparison to healthy controls. All the effect sizes within categories of emotion regulation ability were large, including general emotion regulation ability (Hedges' *g* = −2.04, *p* < 0.001, 95% CI [−2.69, −1.38]), alexithymia (Hedges' *g* = 1.45, *p* < 0.001, 95% CI [0.95, 1.95]), emotional awareness (Hedges' *g* = −0.95, *p* < 0.001, 95% CI [−1.29, −0.61]), clarity (Hedges' *g* = −1.50, *p* < 0.001, 95% CI [−1.86, −1.13]), and tolerance (Hedges' *g* = −1.89, *p* < 0.000, 95% CI [−2.27, −1.50]).

### Participants with remitted MDD

#### Maladaptive emotion regulation strategies

The pooled effect size (Hedges' *g*) for all maladaptive emotion regulation strategies across all studies involving participants with a history of MDD was large (Hedges' *g* = 1.00, *p* < 0.001; 95% CI [0.82, 1.18]). This indicates that although participants were not diagnosed with current depression at the time of the study, they still report applying maladaptive emotion regulation strategies more frequently than healthy adults. More specifically, effect sizes were large for maladaptive emotion regulation strategies of rumination (Hedges' *g* = 1.10, *p* < 0.001, 95% CI [0.90, 1.30] and avoidance (Hedges' *g* = 0.95, *p* < 0.001, 95% CI [9.49, 1.22]), and moderate within suppression (Hedges' *g* = 0.56, *p* < 0.001, 95% CI [0.22, 0.90]).

#### Adaptive emotion regulation strategies

The pooled effect size (Hedges' *g*) for all adaptive emotion regulation strategies across all studies involving participants with a history of MDD was −0.20. However this effect size was not significant (*p* = 0.30, 95% CI [−0.58, 0.18]). Acceptance, problem solving and reappraisal had statistically insignificant effect sizes (see Table 2).

#### General emotion regulation ability

Two of the included studies measured general emotion regulation ability. The results show that participants recovered or in remission of MDD had lower emotion regulation competence than healthy controls, as evident by a moderate effect size (Hedges' *g* = −0.49, *p* < 0.001, 95% CI [−0.68, −0.30]). None of the included studies reported data on alexithymia, emotional awareness, clarity, and tolerance.

### Risk of bias across studies

Egger's regression test (see Tables 1, 2) showed that all analyses but maladaptive emotion regulation strategies and rumination in studies involving current MDD had intercepts that were not significantly different from zero, thereby indicating that, in general, the estimates were not influenced by potential publication bias. However, the Duval and Tweedie's trim and fill procedure indicated that there were some missing studies to the left or right of the mean in specific analyses with studies involving current and remitted MDD. Within studies involving current MDD, studies were missing in analyses involving maladaptive emotion regulation strategies (13 studies left of the mean) with an adjusted effect size of 1.51; avoidance (four studies left of the mean) with an adjusted effect size of 1.03, rumination (six studies left of the mean) with an adjusted effect size of 1.96, acceptance (one study right of the mean) with an adjusted effect size of −0.89, problem solving (one study left of the mean) with an adjusted effect size of −1.13, reappraisal (three studies right of the mean) with an adjusted effect size of −0.54, and finally general emotion regulation ability (one study left of the mean) with an adjusted effect size of −2.18. For studies involving remitted MDD, studies were missing in analyses involving avoidance (one study left of the mean) with an adjusted effect size of 0.83, rumination (two studies right of the mean) with an adjusted effect size of 1.14, and finally adaptive emotion regulation strategies (two studies right of the mean) with an adjusted statistically insignificant effect size of −0.03. In sum, although these analyses indicate some publication bias within some of the meta-analytic syntheses, the adjusted values did not affect the interpretation of the effect sizes.

### Additional analyses

We ran several meta-regression analyses to assess the effect of potential moderators on the effect sizes. Due to limited reporting on potential moderators in the included studies, planned meta-regressions involving avoidance, suppression, reappraisal, acceptance, problem solving, and general emotion regulation abilities were omitted for both current MDD and remitted MDD. For remitted MDD we also had to omit adaptive emotion regulation strategies due to a low number of studies. Consequently, we ran meta-regression analyses to investigate the contribution of moderators on the effect sizes on maladaptive emotion regulation strategies and rumination for current and remitted MDD, and adaptive emotion regulation strategies for current MDD. The results are summarized in Table 3 for individuals with current MDD and Table 4 for individuals with remitted MDD. For participants with current MDD, neither number of previous episodes, nor duration of current episode had an effect on the effect sizes. However, comorbid anxiety was positively associated with maladaptive emotion regulation strategies (β = 0.01, *p* = 0.05) and rumination (β = 0.01, *p* < 0.001), and early age of onset was associated with increased use of maladaptive emotion regulation strategies (β = −0.14, *p* < 0.001).

**Table 3:** Meta-regression with studies including participants with current MDD.

	**Maladaptive emotion regulation strategies**					**Adaptive emotion regulation strategies**					**Rumination**				
	***k***	**β**	**95 % CI**	**SE**	***p***	***k***	**β**	**95 % CI**	**SE**	***p***	***k***	**β**	**95 % CI**	**SE**	***p***
**MODERATORS**															
Number of previous episodes	14	0.01	−0.25, 0.27	0.12	0.96	5	0.19	−0.44, 0.83	0.20	0.40	12	0.02	−0.22, 0.25	0.11	0.86
Comorbid anxiety	25	0.01	0.00, 0.02	0.01	0.05	6	0.03	0.00, 0.06	0.01	0.06	17	0.01	0.00, 0.02	0.00	<0.001
Onset age	9	−0.14	−0.21, −0.07	0.03	<0.001	4	−0.06	−0.17, 0.06	0.03	0.18	6	−0.01	−0.30, 0.29	0.11	0.96

*k indicates number of studies included in the analyses*.

**Table 4:** Meta-regression with studies including participants with remitted MDD.

	**Maladaptive emotion regulation strategies**					**Rumination**				
	***k***	**β**	**95 % CI**	**SE**	***p***	***k***	**β**	**95 % CI**	**SE**	***p***
**MODERATORS**										
Number of previous episodes	14	0.07	−0.12, 0.26	0.09	0.42	10	0.08	−0.10, 0.27	0.08	0.33
Duration remission	6	−0.01	−0.02, 0.00	0.00	0.02	3	–	–	–	–
Comorbid anxiety	9	0.01	−0.03, 0.05	0.02	0.57	7	0.05	0.01, 0.08	0.01	0.02
Onset age	8	0.00	−0.06, 0.06	0.03	0.92	6	0.00	−0.09, 0.08	0.03	0.95

*k indicates number of studies included in the analyses. Where k was lesser than 4, no meta-regression was carried out*.

For participants remitted from MDD, only duration of remission was negatively associated with maladaptive emotion regulation strategies (β = −0.01, *p* = 0.02), and comorbid anxiety had a positive association with rumination (β = 0.05, *p* = 0.02).

## Discussion

The general aim of this systematic review and meta-analysis was to examine and summarize what is known to date about self-reported emotion regulation strategy use and general emotion regulation ability in individuals with current and remitted MDD. The present review adds to earlier efforts by including a systematic literature search and meta-analysis of studies with diagnostically homogeneous groups. As a result, a considerably greater number of studies were identified, reviewed and included, compared to earlier reviews (e.g., Aldao et al., 2010; Liu and Thompson, 2017). Whereas Aldao et al. (2010) included only eight studies with individuals with properly diagnosed MDD, the current study included 72 studies. Moreover, Liu and Thompson (2017) included 15 studies on the emotion regulation strategy of rumination, five on suppression, five on acceptance and seven on reappraisal. In comparison, the current review included 51 studies on rumination, nine on suppression, eight on acceptance and 15 on reappraisal. In sum, our synthesis of the research can be argued to expand the knowledge base on emotion regulation difficulties in MDD.

At the same time, similar patterns of results as in previous reviews emerge. Compared to healthy controls, individuals diagnosed with current MDD report habitually using maladaptive emotion regulation strategies more frequently, and adaptive emotion regulation strategies less frequently. This lends support to the hypothesis that depression is associated with a disposition to use less effective strategies, and difficulties in implementing effective strategies (Joormann and Vanderlind, 2014). Although few included studies measured general emotion regulation ability, the current review provides some indication that individuals with current MDD have difficulties with general emotion regulation in the form of emotional awareness, clarity, and tolerance.

For individuals with remitted MDD, the present review is in line with Joormann and Stanton (2016) and Liu and Thompson (2017) in that these individuals seem to use maladaptive emotion regulation strategies more often than healthy controls. However, no significant difference between individuals remitted from MDD and healthy controls were found in adaptive emotion regulation strategy use. Moreover, results from two studies indicate that remitted MDD individuals have limited general emotion regulation abilities compared to healthy controls. No studies included measures of alexithymia, emotional clarity, awareness or tolerance in individuals with remitted MDD. The meta-analytic results for broader emotion regulation capacities should therefore be regarded as tentative awaiting further research.

Our meta-regression analyses found use of maladaptive emotion regulation strategies for individuals with MDD to be positively associated with comorbid anxiety disorder. This indicates that the presence of anxiety disorders along with depression increases the probability of having difficulties with emotion regulation. Also, age of onset was negatively associated with maladaptive emotion regulation use. This indicates that early debut of the first episode of MDD may be linked to greater problems in managing emotions. Further, comorbid anxiety disorders were positively associated with rumination in both current and remitted MDD. In individuals remitted from MDD, duration of remission was negatively associated with maladaptive emotion regulation strategy use.

### Key findings in trials with current MDD

The results of the systematic review showed that the majority of participants were assessed using SCID-I. The self-reported symptom measure of depression (BDI-II) validated current depression severity. Compared to adaptive emotion regulation strategies, maladaptive emotion regulation strategies were most studied. Within the maladaptive emotion regulation strategies, rumination was the most assessed strategy, included in over half of the eligible studies. In the studies that assessed avoidance, a variety of self-report instruments were applied, indicating a lack of agreement in the field on how to best measure this construct. Considering adaptive emotion regulation strategies, reappraisal was the most studied strategy. Within the strategies acceptance, problem solving and reappraisal, a great diversity of different questionnaires were used, indicating lack of consensus regarding the measurement of these concepts. Only one study reported data from a questionnaire measuring self-compassion. Therefore, self-compassion was omitted from subsequent analyses. A minority of studies included assessments of general emotion regulation ability. Although self-compassion and general emotion regulation abilities may be of theoretic and practical interest, there are still too few empirical investigations of these constructs to warrant valid conclusions about their role in MDD.

The results from the meta-analysis support the hypothesis that individuals with current MDD use more maladaptive emotion regulation strategies compared to healthy controls, as was evident by the large effect sizes obtained within all subcategories of maladaptive emotion regulation strategies. The very large effect size found for rumination supports previous findings (Joormann and Stanton, 2016; Liu and Thompson, 2017), showing that the tendency to dwell on the causes and implications of emotions and emotion eliciting events is a central feature of current MDD. It is also notable that a large effect size was found for avoidance, a strategy often associated with anxiety disorders, but increasingly also shown to be a feature of depression (Brockmeyer et al., 2015). Fewer studies had investigated suppression, but for these, a large effect size was found. The large effect size for suppression supports earlier findings that the use of suppression is associated with increased rumination (Liverant et al., 2011). Moreover, our findings may serve to clarify the role of suppression in MDD, as previous reviews have reported inconsistent results (Joormann and Stanton, 2016; Liu and Thompson, 2017). Taken together, our results indicate that MDD is associated with an inability to disengage from repetitive negative thinking, and attempts to avoid or control unwanted mental and emotional experiences.

Further, our meta-analysis show that people with current MDD report using less adaptive emotion regulation strategies compared to healthy controls. The effect sizes for adaptive emotion regulation strategies were large, with exception of reappraisal which was moderate. This is in line with prior research, as maladaptive emotion regulation strategies like avoidance, rumination and suppression are, in various degrees, negatively correlated with adaptive emotion regulation strategies like acceptance, problem solving and reappraisal (Naragon-Gainey et al., 2017). One reason for this finding may be that implementing adaptive emotion regulation strategies may demand more effort. For example, reappraisal requires a cognitive reframing or change of perspective that can be experienced as too difficult due to depressive mood or other inhibiting factors (Joormann and Stanton, 2016). Interestingly, the finding on reappraisal contrasts findings from experimental studies. For example, in laboratory settings, people with MDD (Ehring et al., 2010) and dysphoria (Quigley and Dobson, 2014) are able to spontaneously use reappraisal to a similar degree as never-depressed controls. Moreover, when individuals with MDD (Millgram et al., 2015) and dysphoria (Quigley and Dobson, 2014) are instructed to use reappraisal, they have no difficulties in doing so, and gain same beneficial reductions in negative affect as healthy controls. This discrepancy in results between self-report and experimental assessments of reappraisal may be a result of different demands of a situation (i.e., simplified laboratory settings vs. complex day-to-day situations) and difficulties in implementing reappraisal if not instructed to do so. Moreover, the presence of an experimenter may have been experienced as supportive, therefore facilitating access to adaptive emotion regulation strategies.

Our findings on adaptive emotion regulation strategies add to prior reviews and meta-analytic findings. Liu and Thompson (2017) found that currently depressed individuals reported less acceptance and reappraisal compared to healthy controls. Aldao et al. (2010) found no association between symptoms of depression and acceptance, and a relative low association between symptoms of depression and reappraisal. Our findings show that people currently diagnosed with MDD were clearly distinguished from healthy controls by their comparatively less frequent use of adaptive emotion regulation strategies. This discrepancy in results may be due to the fact that the prior meta-analysis by Aldao et al. (2010) was based on fewer studies on the strategies of acceptance and reappraisal, in addition to including more heterogeneous samples with regards to diagnostic status. In our study, measurement error may have been reduced as a result of using a more homogenous population (properly diagnosed MDD), in addition to achieving more precise estimates due to the inclusion of more studies (Borenstein et al., 2009).

Our results further indicate that participants with current MDD have limited general emotion regulation abilities, and report having problems with emotional awareness, understanding, clarity, and tolerance. Thus, currently depressed individuals have limited capacities to identify and describe emotions, exhibit less awareness toward emotions, and tolerate emotions to a lesser extent compared to healthy controls. This finding should, however, be interpreted with caution, because the effect sizes are based on a limited amount of studies (*k* = 7). Nevertheless, the findings lend some support to the notion that people with MDD have less knowledge and awareness of their emotion, in addition to being more easily overwhelmed by emotions. Seen in connection with findings on specific emotion regulation strategies, the results support the notion that general emotion regulation abilities may be a set of complementary processes that influence the types of emotion regulation strategies the individuals may engage in Tull and Aldao (2015). Poor general emotion regulation abilities may lead to more frequent and inflexible use of maladaptive emotion regulation strategies like avoidance, suppression and rumination (Jeffries et al., 2016; Naragon-Gainey et al., 2017).

The finding that depressed individuals use more maladaptive emotion regulation strategies and less adaptive emotion regulation strategies may not be a surprising one. It could be that these deficiencies are due to the depressed state itself. Depression increases maladaptive cognitive and emotional processes, as well as peoples' tendencies to evaluate themselves negatively on self-report measures. As such, it cannot be determined to what extent these findings reflect trait level strategy use. However, empirical investigations show that individuals with MDD have executive function deficits (Joormann and Vanderlind, 2014). These particular deficits may render the use of adaptive emotion regulation strategies more difficult than maladaptive ones. For example, the difficulties in disengaging ones attention from repetitive negative thinking (i.e., rumination) will prohibit the use of more flexible and constructive emotion regulation strategies, and result in prolonged sad mood. Likewise, difficulties in excluding irrelevant negative information from working memory (i.e., limited cognitive control), may lead to increased maladaptive emotion regulation strategy use (Joormann and Vanderlind, 2014). However, the direction of the relationship between executive functioning and emotion regulation is unclear. For instance, results from one study show that instructed rumination affect working memory functioning (Watkins and Brown, 2002), indicating that the tendency to ruminate may be a contributor to executive dysfunction in individuals with MDD. In sum, the effect sizes indicate that difficulties in emotion regulation are prominent in current depression, pointing to the potential therapeutic utility of addressing these processes in order to alleviate the disorder.

Our meta-regression analyses found an association between comorbid anxiety and maladaptive emotion regulation use and rumination. Thus, the higher proportion of participants with comorbid anxiety disorders in a sample, the more general maladaptive emotion regulation use and rumination was reported. This finding indicates that emotion regulation can be seen as a transdiagnostic factor, in that the overreliance on maladaptive and under-reliance on adaptive strategies can be seen as giving rise to various diagnostic categories of psychological distress (Fernandez et al., 2016). It is also indicative of a cumulative effect, in that the presence of comorbid states are positively associated with habitual use of maladaptive emotion regulation strategies. Finally, early onset age was positively associated with higher maladaptive emotion regulation use. The younger the individuals were when experiencing the first episode of MDD, the more use of maladaptive emotion regulation strategies was reported. Early onset of MDD can be associated with the exposure of early life stress (Molnar et al., 2001). Overwhelming experiences in early age may lead to increased habitual use of maladaptive emotion regulation strategies. Thus, early onset may predispose individuals for later psychopathology due to difficulties in emotion regulation. In sum, our moderator analyses indicate that more complex problems with larger duration are associated with poorer emotion management. This may point to a particularly vulnerable group whose difficulties in emotion regulation should be addressed.

### Key findings in trials with remitted MDD

All included studies with participants with remitted MDD used the SCID-I in order to assess former and current psychiatric diagnoses. The low mean score of the self-reported symptom measure of depression (BDI-II) indicated absence of current depression. Taken together, absence of diagnoses and BDI-II scores in the normal range indicates that these individuals were actually non-depressed at the point of participating in the studies. This is important, at it points to the presence of continuing difficulties in emotion regulation after the depressive episode has remitted. As was evident in trials with participants with current MDD, maladaptive emotion regulation strategies were most common in comparison to adaptive emotion regulation strategies. Rumination was the most studied maladaptive emotion regulation strategy, and reappraisal the most studied adaptive emotion regulation strategy. Only two studies included assessment of general emotion regulation ability.

The large effect size for rumination in remitted MDD supports prior findings (Joormann and Stanton, 2016; Liu and Thompson, 2017) that individuals with remitted MDD are more prone to negative repetitive thinking compared to healthy controls. Further, the present study also found a large effect size for avoidance and a moderate effect size for suppression. The latter finding clarifies that individuals with remitted MDD are more likely than healthy controls to inhibit the expression or experience of emotions. Taken together, although the participants in the studies were not currently depressed, they employed maladaptive emotion regulation strategies to a greater extent than healthy controls. Thus, the use of maladaptive emotion regulation strategies may be a latent vulnerability that remains also after the depressive symptoms are alleviated. Given the recurrent nature of MDD (Mueller et al., 1999), the tendency to habitually use maladaptive emotion regulation strategies could be one factor that leads to the development of new depressive episodes. Longitudinal studies confirm that self-reported use of maladaptive emotion regulation strategies is associated with future relapses of MDD (Aldao and Nolen-Hoeksema, 2012; Berking et al., 2014). Furthermore, as former studies indicate that people have positive beliefs about applying rumination when managing emotions (Watkins and Moulds, 2005b), these strategies may be applied to cope with negative emotions after remission from MDD. Consequently, the increased use of such strategies may be a risk factor that may lead to future episodes of MDD. Alternatively, it may be that the overreliance on maladaptive strategies may reflect an underlying deficit in executive functioning. Our finding may be seen in relation to prior reviews (Hammar and Årdal, 2009) and meta-analyses (Rock et al., 2013) showing that attentional and executive deficits continue after remission from MDD.

With regard to adaptive emotion regulation strategies, no significant difference was found between individuals with remitted MDD and healthy controls. However, due to the limited number of studies that assessed acceptance (*k* = 2) and problem solving (*k* = 1) these effect sizes should be interpreted with caution, as the effect sizes might have been significant if more studies reported these outcomes. Nevertheless, we found no difference between remitted MDD individuals and healthy controls. This finding may be valid as it was based on five studies and a considerable number of participants, and support the findings of previous reviews (Joormann and Stanton, 2016; Liu and Thompson, 2017). As suggested by Liu and Thompson (2017), reappraisal may be a maintaining factor of MDD, but not a risk factor for new episodes of MDD. On this basis, one could hypothesize that vulnerability to depressive relapse lie in certain persistent dysfunctional habits of emotion regulation rather than in the absence of good habits. That is, the attempts of the individuals to use strategies of acceptance, constructive problem solving, and reappraisal may be hindered by co-occurring processes of avoidance, rumination, and suppression. This may in turn point to the utility of addressing these hindrances in relapse prevention as the training of positive skills may not in itself be sufficient.

With respect to general emotion regulation ability, only two studies reported outcomes giving a negative moderate effect size. This indicates that individuals with remitted MDD have limited general emotion regulation abilities. In line with former evidence that indicate general emotion regulation abilities are considered a factor for developing new depressive episodes (Saarijärvi et al., 2001; Ehring et al., 2008; Ebert et al., 2017), our findings support the notion that people with remitted MDD still have limited ability to be aware of their own emotions, perceive them with clarity and tolerate them. This broader deficiency in emotion regulation may be seen as a general vulnerability factor that needs to be better understood.

In terms of moderators, we found, similar to the current MDD studies, that comorbid anxiety is associated with increased self-reported rumination. Interestingly, duration of remission was positively associated with lesser maladaptive emotion regulation strategy use. That is, the longer people have been well from depression, the less unproductive strategies they were liable to use. This could indicate a waning of their vulnerability to relapse as time goes by, indicating that the longer one can postpone the reoccurrence of depression, the better the person will be off. Another possibility is that maladaptive strategies simply subside over time as the residual depressive symptoms subside.

### Implications

The results of the present review and meta-analysis have implications for future research and clinical trials. For future studies on self-reported emotion regulation, our findings point to the need for improving the quality and reporting of diagnostic procedures. A substantial number of potential eligible studies did not report on diagnostic procedures or reported methodologically questionable procedures not involving structured interviews. Future studies should make sure that both clinical groups and normative controls are properly assessed. The potential moderators in the reviewed studies were reported in a sparse and unsystematic way. Researchers are advised to report on potential moderators (i.e., personality disorders, illness onset age), enabling more informative analyses in future reviews. Further, the investigation of self-reported emotion regulation difficulties associated with emotional awareness, clarity and tolerance should be addressed in both current and remitted MDD. These broader capacities may constitute a foundation for more specific regulatory strategies that could further our understanding of the emotion regulation process. In the meantime, subsequent meta-analyses may investigate these capacities and their associations with self-reported symptoms of depression. This will probably lead to more included studies, and consequently gain more insight on the association between MDD and general emotion regulation abilities.

Future studies should also explore the habitual use of adaptive emotion regulation strategies, as such evidence is needed to make valid conclusions involving individuals with remitted MDD. More specifically, more studies exploring self-compassion and problem solving in properly diagnosed individuals with current and past MDD are called upon.

With respect to clinical implications, the finding that maladaptive emotion regulation strategies are prominent in a current episode of MDD, and also persist after remission from depression shed light on the importance of maladaptive emotion regulation strategies as a target of psychotherapy. In the recent years, interventions have been developed specifically to enhance emotion regulation in anxiety disorders (Mennin, 2006), personality disorders (Gratz and Gunderson, 2006), and inpatient treatment for MDD (Berking et al., 2013). More studies are needed to further explore the effect of such studies on emotion regulation strategies and ability, with follow-up assessment to investigate the relapse rate of the disorders.

Further, our findings indicate that onset age of depression should be of interest in tailoring interventions and therapies, as this may have an impact on emotion regulation difficulties. Previous studies have shown that treatment of depression using Mindfulness-Based Cognitive Therapy (MBCT) have better effect on individuals with an early onset in comparison to individuals with later onset (Williams et al., 2014). Moreover, prevention programs and interventions aimed at younger populations should address emotion regulation and management to further protect these populations against onset and recurrence of MDD.

### Limitations of the literature

In our systematic review, several limitations were found in the literature. First, close to a hundred studies were excluded due to lack of structured diagnostic assessment or unclear reporting of diagnostic procedures. This means that potentially valuable knowledge about emotion regulation in MDD is lost, as it is impossible to determine whether participants actually belong to the diagnostic category in question. Regarding the psychometric instruments used in the included studies, it was evident that there is a lack of agreement of scales to assess emotion regulation. It could also be the case that the present categories mask important variation with regard to emotion regulation. For instance, the category of suppression includes both expressive and experiential modes of processing, which have been indicated to be differentially related to positive outcomes (Webb et al., 2012). Also, a very limited number of studies included adaptive emotion regulation strategies and general emotion regulation abilities as outcomes. At present, the research has focused on a narrow subset of emotion regulation processes, mainly maladaptive ones. This limits the conclusions that can be drawn. Further, it is a limitation that current self-report instruments do not capture the complexity of the emotion regulation process. The field of emotion regulation research at large is progressing toward more complex conceptualizations of affective dynamics. This includes a more refined consideration of contextual factors (Aldao et al., 2015), as well as the importance of individual flexibility in applying regulatory strategies (Sheppes et al., 2015). Finally, few studies reported potential moderators that can contribute to emotion regulation difficulties. As a result, relevant moderator analyses can as of yet not be carried out, and the current moderator analyses had low sample sizes, and are thus less informative.

### Limitations of this review

Although the present review identified and selected eligible studies though a systematic literature search and extensive review of reference lists, potentially eligible studies may not have been detected in the search process. Also, our meta-analytic summaries of general emotion regulation abilities were based on a limited number of studies, thus questioning the validity of these findings. Another limitation of this review may be the selection of strategies. Although we chose to base our study on an earlier review by Aldao et al. (2010), a variety of emotion regulation strategies have been suggested (Augustine and Hemenover, 2009; Webb et al., 2012). Other strategies that were not included in our review may be of importance, such as various ways of upregulating positive affect. However, as Liu and Thompson (2017) showed in their review, strategies like positive rumination, dampening and savoring have been comparatively less researched compared to the “well-established” strategies of acceptance, problem solving and reappraisal. Other emotion regulation strategies may nevertheless constitute an important facet of skillful emotion regulation.

Another limitation of our review is that it is based on self-report questionnaires. The use of self-report questionnaires is prone to several methodological issues, including being dependent on individuals' self-perspective and psychological mindedness. It is unclear to what extent people with current MDD are able to accurately assess their habitual use of ER strategies and abilities, given the state that they are in. Currently depressed individuals may have distorted views of themselves, thus resulting in a negative bias in self-reports colored by their negative or unflattering self-perceptions. However, this was partly resolved by including studies with individuals with remitted MDD, as these individuals reported non-clinical levels of depressive symptoms. Also, people may regulate emotions in ways that are habitual or highly automated, making these strategies less accessible to introspection and explicit self-report. It can also be argued that self-report questionnaires cannot capture the complex concept of emotion regulation. Emotions are multifaceted phenomena involving both experiential, behavioral and somatic domains, which cannot be covered by self-reported questionnaires alone (Gross, 2015). Although the reviews of Joormann and Stanton (2016) and Liu and Thompson (2017) did include experimental studies of emotion regulation, neither were based on systematic literature searches, thus potentially leaving out several eligible studies. Future systematic reviews would benefit from integrating data from behavioral or performance based measures of emotion regulation. Such measures avoid some of the problems of self-report instruments, and might yield more accurate estimates of emotion regulation.

## Conclusion

Individuals with both current and remitted MDD have difficulties with emotion regulation. Currently and remitted MDD individuals report using more maladaptive emotion regulation strategies compared to healthy controls. Currently depressed individuals report using less adaptive emotion regulation strategies, and report having limited general emotion regulation abilities, including emotional awareness, clarity, and tolerance. Due to a limited number of studies involving adaptive emotion regulation strategies in persons with remitted MDD, the null results of acceptance and problem solving should be interpreted with caution. However, the studies that include reappraisal suggest that there is no difference between individuals with remitted MDD and healthy controls. Moreover, the studies indicate that individuals with remitted MDD have limited general emotion regulation abilities compared to healthy controls, although this result is also based on a limited number of studies. Comorbid anxiety influences maladaptive emotion regulation in individuals with both current and remitted MDD, and early onset of MDD could be an important factor influencing maladaptive emotion regulation. Finally, duration of remission seems to be a protective factor of future relapse, as longer duration of remission was associated with lesser maladaptive emotion regulation use.

In sum, these findings can inform future research in terms of securing adequate diagnostic procedures, including individuals with remitted MDD, assessing general emotion regulation abilities, reporting important moderator variables, and utilizing laboratory and performance based measures. Clinically, the presence of emotion regulation deficiencies in both ongoing and former depression points to the usefulness of addressing these issues both in treatment and relapse prevention.

## Author contributions

EV designed the study, wrote the protocol of the study, conducted the literature search, read initial abstracts, extracted data from potential eligible studies, conducted the statistical analyses and wrote the first draft of the manuscript. JV contributed with supervision in protocol, design and manuscript writing, concrete suggestions for change in the manuscript and extracted data from potential eligible studies. MN contributed with supervision in statistical analyses, concrete suggestions for statistical analyses, and co-writing of the statistical analyses paragraph. ES contributed with supervision in protocol, design and manuscript writing, and concrete suggestions for change in the manuscript.

### Conflict of interest statement

The authors declare that the research was conducted in the absence of any commercial or financial relationships that could be construed as a potential conflict of interest.
