# Supplementary material for: Emotion Regulation in Current and Remitted Depression: A Systematic Review and Meta-Analysis
Source: Front Psychol. 2018 May 18;9:756. doi: 10.3389/fpsyg.2018.00756 (PMC5968125; doi:10.3389/fpsyg.2018.00756)
Supplement: Supplementary file 1 [file Table_1.pdf]

Appendix A
References of included studies:

Abravanel, B. T., & Sinha, R. (2015). Emotion dysregulation mediates the relationship between lifetime cumulative adversity and depressive symptomatology. *Journal of Psychiatric Research, 61,* 89-96. doi:10.1016/j.jpsychires.2014.11.012

Aker, M., Harmer, C., & Landro, N. I. (2014). More rumination and less effective emotion regulation in previously depressed women with preserved executive functions. *BMC Psychiatry, 14,* 334. Retrieved from http://www.biomedcentral.com/1471-244X/14/334

Alderman, B. L., Olson, R. L., Bates, M. E., Selby, E. A., Buckman, J. F., Brush, C. J., . . . Shors, T. J. (2015). Rumination in major depressive disorder is associated with impaired neural activation during conflict monitoring. *Frontiers in Human Neuroscience, 9,* 269. doi:10.3389/fnhum.2015.00269

Batmaz, S., Ulusoy Kaymak, S., Kocbiyik, S., & Turkcapar, M. H. (2014). Metacognitions and emotional schemas: A new cognitive perspective for the distinction between unipolar and bipolar depression. *Comprehensive Psychiatry, 55*(7), 1546–1555. doi:10.1016/j.comppsych.2014.05.016

Beblo, T., Fernando, S., Klocke, S., Griepenstroh, J., Aschenbrenner, S., & Driessen, M. (2012). Increased suppression of negative and positive emotions in major depression. *Journal of Affective Disorders, 141*(2-3), 474-479. doi:10.1016/j.jad.2012.03.019

Belleau, E. L., Taubitz, L. E., & Larson, C. L. (2015). Imbalance of default mode and regulatory networks during externally focused processing in depression. *Soc Cogn Affect Neurosci, 10*(5), 744-751. doi:10.1093/scan/nsu117

Bensaeed, S., Jolfaei, A. G., Jomehri, F., & Moradi, A. (2014). The relationship between major depressive disorder and personality traits. *Iranian Journal of Psychiatry, 9*(1), 37-41. Retrieved from https://www.ncbi.nlm.nih.gov/pmc/articles/PMC4277606/

Brockmeyer, T., Bents, H., Holtforth, M. G., Pfeiffer, N., Herzog, W., & Friederich, H.-C. (2012). Specific emotion regulation impairments in major depression and anorexia nervosa. *Psychiatry Research, 200*(2-3), 550-553. doi:10.1016/j.psychres.2012.07.009

Brockmeyer, T., Holtforth, M. G., Pfeiffer, N., Backenstrass, M., Friederich, H.-C., & Bents, H. (2012). Mood regulation expectancies and emotion avoidance in depression vulnerability. *Personality and Individual Differences, 53*(3), 351-354. doi:10.1016/j.paid.2012.03.018

Brockmeyer, T., Kulessa, D., Hautzinger, M., Bents, H., & Backenstrass, M. (2015). Differentiating early-onset chronic depression from episodic depression in terms of cognitive-behavioral and emotional avoidance. *Journal of Affective Disorders, 175,* 418-423. doi:10.1016/j.jad.2015.01.045

Chan, J. C., Davey, G. C., & Brewin, C. R. (2013). Understanding depressive rumination from a mood-as-input perspective: Effects of stop-rule manipulation. *Behaviour Research and Therapy, 51*(6), 300-306. doi:10.1016/j.brat.2013.02.007

Clark, L., Dombrovski, A. Y., Siegle, G. J., Butters, M. A., Shollenberger, C. L., Sahakian, B. J., & Szanto, K. (2011). Impairment in risk-sensitive decision-making in older suicide attempters with depression. *Psychology and Aging, 26*(2), 321-330. doi:10.1037/a0021646

Cooney, R. E., Joormann, J., Eugene, F., Dennis, E. L., & Gotlib, I. H. (2010). Neural correlates of rumination in depression. *Cognitive, Affective & Behavioral Neuroscience, 10*(4), 470-478. doi:10.3758/CABN.10.4.470

Deveney, C. M., & Deldin, P. (2006). A preliminary investigation of cognitive flexibility for emotional information in major depressive disorder and non-psychiatric controls. *Emotion, 6*(3), 429-437. doi:10.1037/1528-3542.6.3.429

Dillon, D. G., & Pizzagalli, D. A. (2013). Evidence of successful modulation of brain activation and subjective experience during reappraisal of negative emotion in unmedicated depression. *Psychiatry Research: Neuroimaging, 212*(2), 99-107. doi:10.1016/j.pscychresns.2013.01.001

Donaldson, C., & Lam, D. (2004). Rumination, mood and social problem-solving in major depression. *Psychological Medicine, 34*(7), 1309-1318. doi:10.1017/S0033291704001904

Ehret, A. M., Joormann, J., & Berking, M. (2015). Examining risk and resilience factors for depression: The role of self-criticism and self-compassion. *Cognition & Emotion, 29*(8), 1496-1504. doi:10.1080/02699931.2014.992394

Ekinci, O., Albayrak, Y., & Ekinci, A. E. (2012). Temperament and character in euthymic major depressive disorder patients: The effect of previous suicide attempts and psychotic mood episodes. *Psychiatry Investigation, 9*(2), 119-126. doi:10.4306/pi.2012.9.2.119

Farb, N. A. S., Anderson, A. K., Bloch, R. T., & Segal, Z. V. (2011). Mood-linked responses in medial prefrontal cortex predict relapse in patients with recurrent unipolar depression. *Biological Psychiatry, 70*(4), 366-372. doi:10.1016/j.biopsych.2011.03.009

Farmer, A., Mahmood, A., Redman, K., Harris, T., Sadler, S., & McGuffin, P. (2003). A sib-pair study of the Temperament and Character Inventory scales in major depression. *Archives of General Psychiatry, 60*(5), 490-496. doi:10.1001/archpsyc.60.5.490

Fernando, S. C., Beblo, T., Schlosser, N., Terfehr, K., Otte, C., Lowe, B., . . . Wingenfeld, K. (2014). The impact of self-reported childhood trauma on emotion regulation in borderline personality disorder and major depression. *Journal of Trauma & Dissociation, 15*(4), 384-401. doi:10.1080/15299732.2013.863262

Fladung, A., Baron, U., Gunst, I., & Kiefer, M. (2010). Cognitive reappraisal modulates performance following negative feedback in patients with major depressive disorder. *Psychological Medicine, 40*(10), 1703-1710. doi:10.1017/S0033291709992170

Fletcher, K., Parker, G. B., & Manicavasagar, V. (2013). Coping profiles in bipolar disorder. *Comprehensive Psychiatry, 54*(8), 1177-1184. doi:10.1016/j.comppsych.2013.05.011

Gibbs, L. M., Dombrovski, A. Y., Morse, J., Siegle, G. J., Houck, P. R., & Szanto, K. (2009). When the solution is part of the problem: Problem solving in elderly suicide attempters. *International Journal of Geriatric Psychiatry, 24*(12), 1396-1404. doi:10.1002/gps.2276

Halvorsen, M., Hagen, R., Hjemdal, O., Eriksen, M. S., Sorli, A. J., Waterloo, K., . . . Wang, C. E. (2015). Metacognitions and thought control strategies in unipolar major depression: A comparison of currently depressed, previously depressed, and never-depressed individuals. *Cognitive Therapy and Research, 39*(1), 31-40. doi:10.1007/s10608-014-9638-4

Halvorsen, M., Wang, C. E., Richter, J., Myrland, I., Pedersen, S. K., Eisemann, M., & Waterloo, K. (2009). Early maladaptive schemas, temperament and character traits in clinically depressed and previously depressed subjects. *Clinical Psychology & Psychotherapy, 16*(5), 394-407. doi:10.1002/cpp.618

Hamilton, J., Furman, D. J., Chang, C., Thomason, M. E., Dennis, E., & Gotlib, I. H. (2011). Default-mode and task-positive network activity in major depressive disorder: Implications for adaptive and maladaptive rumination. *Biological Psychiatry, 70*(4), 327-333. doi:10.1016/j.biopsych.2011.02.003

Hsu, S.-C., Liu, C.-Y., & Hsiao, M.-C. (2007). A comparison of the Tridimensional Personality Questionnaire in premenstrual dysphoric disorder and major depressive disorder. *Comprehensive Psychiatry, 48*(4), 366-370. doi:10.1016/j.comppsych.2007.02.006

Huffziger, S., Ebner-Priemer, U., Zamoscik, V., Reinhard, I., Kirsch, P., & Kuehner, C. (2013). Effects of mood and rumination on cortisol levels in daily life: An ambulatory assessment study in remitted depressed patients and healthy controls. *Psychoneuroendocrinology, 38*(10), 2258-2267. doi:10.1016/j.psyneuen.2013.04.014

Johnson, M. K., Nolen-Hoeksema, S., Mitchell, K. J., & Levin, Y. (2009). Medial cortex activity, self-reflection and depression. *Soc Cogn Affect Neurosci, 4*(4), 313-327. doi:10.1093/scan/nsp022

Joormann, J., Dkane, M., & Gotlib, I. H. (2006). Adaptive and Maladaptive Components of Rumination? Diagnostic Specificity and Relation to Depressive Biases. *Behavior Therapy, 37*(3), 269-280. doi:10.1016/j.beth.2006.01.002

Joormann, J., & Gotlib, I. H. (2008). Updating the contents of working memory in depression: Interference from irrelevant negative material. *J Abnorm Psychol, 117*(1), 182-192. doi:10.1037/0021-843X.117.1.182

Joormann, J., & Gotlib, I. H. (2010). Emotion regulation in depression: Relation to cognitive inhibition. *Cognition and Emotion, 24*(2), 281-298. doi:10.1080/02699930903407948

Joormann, J., Levens, S. M., & Gotlib, I. H. (2011). Sticky thoughts: Depression and rumination are associated with difficulties manipulating emotional material in working memory. *Psychological Science, 22*(8), 979-983. doi:10.1177/0956797611415539

Joormann, J., Nee, D. E., Berman, M. G., Jonides, J., & Gotlib, I. H. (2010). Interference resolution in major depression. *Cognitive, Affective & Behavioral Neuroscience, 10*(1), 21-33. doi:10.3758/CABN.10.1.21

Kircanski, K., Thompson, R. J., Sorenson, J. E., Sherdell, L., & Gotlib, I. H. (2015). Rumination and Worry in Daily Life. *Clinical Psychological Science, 3*(6), 926-939. doi:10.1177/2167702614566603

Koch, J., & Exner, C. (2015). Selective attention deficits in obsessive-compulsive disorder: The role of metacognitive processes. *Psychiatry Research, 225*(3), 550-555. doi:10.1016/j.psychres.2014.11.049

Lau, M. A., Christensen, B. K., Hawley, L. L., Gemar, M. S., & Segal, Z. V. (2007). Inhibitory deficits for negative information in persons with major depressive disorder. *Psychological Medicine, 37*(9), 1249-1259. doi:10.1017/S0033291707000530

Lee, S., Kim, S. J., Park, J. E., Cho, S.-J., Cho, I. H., & Lee, Y. J. (2012). Biogenetic temperament and character in insomnia and depression. *Journal of Psychosomatic Research, 72*(5), 383-387. doi:10.1016/j.jpsychores.2012.01.016

Levens, S. M., Muhtadie, L., & Gotlib, I. H. (2009). Rumination and impaired resource allocation in depression. *J Abnorm Psychol, 118*(4), 757-766. doi:10.1037/a0017206

Mandell, D., Siegle, G. J., Shutt, L., Feldmiller, J., & Thase, M. E. (2014). Neural substrates of trait ruminations in depression. *J Abnorm Psychol, 123*(1), 35-48. doi:10.1037/a0035834

Marchesi, C., Bertoni, S., Cantoni, A., & Maggini, C. (2008). Is alexithymia a personality trait increasing the risk of depression? A prospective study evaluating alexithymia before, during and after a depressive episode. *Psychological Medicine, 38*(12), 1717-1722. doi:10.1017/s0033291708003073

Marchesi, C., Ossola, P., Tonna, M., & De Panfilis, C. (2014). The TAS-20 more likely measures negative affects rather than alexithymia itself in patients with major depression, panic disorder, eating disorders and substance use disorders. *Comprehensive Psychiatry, 55*(4), 972-978. doi:10.1016/j.comppsych.2013.12.008

Meiran, N., Diamond, G. M., Toder, D., & Nemets, B. (2011). Cognitive rigidity in unipolar depression and obsessive compulsive disorder: Examination of task switching, Stroop, working memory updating and post-conflict adaptation. *Psychiatry Research, 185*(1-2), 149-156. doi:10.1016/j.psychres.2010.04.044

Minaya, O., & Fresan, A. (2009). Anxiety disorders comorbidity in first-episode depressed patients: Personality differences based on the Temperament and Character Inventory. *Personality and Individual Differences, 47*(5), 522-526. doi:10.1016/j.paid.2009.05.006

Nery, F. G., Hatch, J. P., Nicoletti, M. A., Monkul, E., Najt, P., Matsuo, K., . . . Soares, J. C. (2009). Temperament and character traits in major depressive disorder: Influence of mood state and recurrence of episodes. *Depression and Anxiety, 26*(4), 382-388. doi:10.1002/da.20478

Nowakowska, C., Strong, C. M., Santosa, C. M., Wang, P. W., & Ketter, T. A. (2005). Temperamental commonalities and differences in euthymic mood disorder patients, creative controls, and healthy controls. *Journal of Affective Disorders, 85*(1-2), 207-215. doi:10.1016/j.jad.2003.11.012

O'Kearney, R., & Parry, L. (2014). Comparative physiological reactivity during script-driven recall in depression and posttraumatic stress disorder. *J Abnorm Psychol, 123*(3), 523-532. doi:10.1037/a0037326

Ottaviani, C., Shahabi, L., Tarvainen, M., Cook, I., Abrams, M., & Shapiro, D. (2014). Cognitive, behavioral, and autonomic correlates of mind wandering and perseverative cognition in major depression. *Frontiers in Neuroscience, 8,* 433. doi:10.3389/fnins.2014.00433

Ottenbreit, N. D., Dobson, K. S., & Quigley, L. (2014). An examination of avoidance in major depression in comparison to social anxiety disorder. *Behaviour Research and Therapy, 56,* 82-90. doi:10.1016/j.brat.2014.03.005

Pearson, K. A., Watkins, E. R., Mullan, E. G., & Moberly, N. J. (2010). Psychosocial correlates of depressive rumination. *Behaviour Research and Therapy, 48*(8), 784-791. doi:10.1016/j.brat.2010.05.007

Pu, S., Nakagome, K., Yamada, T., Yokoyama, K., Matsumura, H., Mitani, H., . . . Kaneko, K. (2012). The relationship between the prefrontal activation during a verbal fluency task and stress-coping style in major depressive disorder: A near-infrared spectroscopy study. *Journal of Psychiatric Research, 46*(11), 1427-1434. doi:10.1016/j.jpsychires.2012.08.001

Regenbogen, C., Kellermann, T., Seubert, J., Schneider, D. A., Gur, R. E., Derntl, B., . . . Habel, U. (2015). Neural responses to dynamic multimodal stimuli and pathology-specific impairments of social cognition in schizophrenia and depression. *British Journal of Psychiatry, 206*(3), 198-205. doi:10.1192/bjp.bp.113.143040

Remmers, C., Topolinski, S., Dietrich, D. E., & Michalak, J. (2015). Impaired intuition in patients with major depressive disorder. *British Journal of Clinical Psychology, 54*(2), 200-213. doi:10.1111/bjc.12069

Riso, L. P., du Toit, P. L., Blandino, J. A., Penna, S., Dacey, S., Duin, J. S., . . . Ulmer, C. S. (2003). Cognitive aspects of chronic depression. *Journal of Abnormal Psychology, 112*, 72-80. doi:10.1037/0021-843X.112.1.72

Sasayama, D., Hori, H., Teraishi, T., Hattori, K., Ota, M., Matsuo, J., . . . Kunugi, H. (2011). Difference in temperament and character inventory scores between depressed patients with bipolar II and unipolar major depressive disorders. *Journal of Affective Disorders, 132*(3), 319-324. doi:10.1016/j.jad.2011.03.009

Scherrer, M. C., Dobson, K. S., & Quigley, L. (2014). Predictors of self-reported negative mood following a depressive mood induction procedure across previously depressed,

currently anxious, and control individuals. *British Journal of Clinical Psychology, 53*(3), 348-368. doi:10.1111/bjc.12053
Schiller, C. E., Minkel, J., Smoski, M. J., & Dichter, G. S. (2013). Remitted major depression is characterized by reduced prefrontal cortex reactivity to reward loss. *Journal of Affective Disorders, 151*(2), 756-762. doi:10.1016/j.jad.2013.06.016
Sigmon, S. T., Pells, J. J., Schartel, J. G., Hermann, B. A., Edenfield, T. M., LaMattina, S. M., . . . Whitcomb-Smith, S. R. (2007). Stress reactivity and coping in seasonal and nonseasonal depression. *Behaviour Research and Therapy, 45*(5), 965-975. doi:10.1016/j.brat.2006.07.016
Svaldi, J., Griepenstroh, J., Tuschen-Caffier, B., & Ehring, T. (2012). Emotion regulation deficits in eating disorders: A marker of eating pathology or general psychopathology? *Psychiatry Research, 197*(1-2), 103-111. doi:10.1016/j.psychres.2011.11.009
Thompson, R. J., Mata, J., Jaeggi, S. M., Buschkuehl, M., Jonides, J., & Gotlib, I. H. (2010). Maladaptive coping, adaptive coping, and depressive symptoms: Variations across age and depressive state. *Behaviour Research and Therapy, 48*(6), 459-466. doi:10.1016/j.brat.2010.01.007
Ubl, B., Kuehner, C., Kirsch, P., Ruttorf, M., Flor, H., & Diener, C. (2015). Neural reward processing in individuals remitted from major depression. *Psychological Medicine, 45*(16), 3549-3558. doi:10.1017/S0033291715001452
Vorontsova, N., Garety, P., & Freeman, D. (2013). Cognitive factors maintaining persecutory delusions in psychosis: The contribution of depression. *J Abnorm Psychol, 122*(4), 1121-1131. doi:10.1037/a0034952
Watkins, E., & Baracaia, S. (2002). Rumination and social problem-solving in depression. *Behaviour Research and Therapy, 40*(10), 1179-1189. doi:10.1016/S0005-7967%2801%2900098-5
Watkins, E., & Brown, R. (2002). Rumination and executive function in depression: An experimental study. *Journal of Neurology, Neurosurgery & Psychiatry, 72*(3), 400-402. doi:10.1136/jnnp.72.3.400
Watkins, E., & Moulds, M. (2005). Positive beliefs about rumination in depression--A replication and extension. *Personality and Individual Differences, 39*(1), 73-82. doi:10.1016/j.paid.2004.12.006
Watkins, E. R., & Moulds, M. L. (2009). Thought control strategies, thought suppression, and rumination in depression. *International Journal of Cognitive Therapy, 2*(3), 235-251. doi:10.1521/ijct.2009.2.3.235
Watson, L., Berntsen, D., Kuyken, W., & Watkins, E. (2013). Involuntary and voluntary autobiographical memory specificity as a function of depression. *Journal of Behavior Therapy and Experimental Psychiatry, 44*(1), 7-13. doi:10.1016/j.jbtep.2012.06.001
Wolkenstein, L., Zwick, J. C., Hautzinger, M., & Joormann, J. (2014). Cognitive emotion regulation in euthymic bipolar disorder. *Journal of Affective Disorders, 160*, 92-97. doi:10.1016/j.jad.2013.12.022
Zamoscik, V., Huffziger, S., Ebner-Priemer, U., Kuehner, C., & Kirsch, P. (2014). Increased involvement of the parahippocampal gyri in a sad mood predicts future depressive symptoms. *Soc Cogn Affect Neurosci, 9*(12), 2034-2040. doi:10.1093/scan/nsu006
Zaninotto, L., Souery, D., Calati, R., Di Nicola, M., Montgomery, S., Kasper, S., . . . Janiri, L. (2015). Temperament and character profiles in bipolar I, bipolar II and major depressive disorder: Impact over illness course, comorbidity pattern and psychopathological features of depression. *Journal of Affective Disorders, 184*, 51-59. doi:10.1016/j.jad.2015.05.036

Zetsche, U., D'Avanzato, C., & Joormann, J. (2012). Depression and rumination: Relation to components of inhibition. *Cognition and Emotion, 26*(4), 758-767. doi:10.1080/02699931.2011.613919
