# Supplementary material for: Emotion Regulation in Current and Remitted Depression: A Systematic Review and Meta-Analysis
Source: Front Psychol. 2018 May 18;9:756. doi: 10.3389/fpsyg.2018.00756 (PMC5968125; doi:10.3389/fpsyg.2018.00756)
Supplement: Supplementary file 2 [file Table_2.pdf]

Appendix B: Table B.1 (current MDD) and B.2 (MDD in remission) with study characteristics

Table B.1

Study characteristics - studies involving patients with current Major Depressive Disorder.

	MDD Sample		Healthy control sample			
Study (year)	N (% female)	M age (SD)	N (% female)	M age (SD)	Moderator(s)	Emotion regulation questionnaire(s)
Alderman et al. (2015)	33 (72 %)	20.7 (2.9)	36 (67 %)	21 (3.1)	Comorbid anxiety = 18 %	RRS-DEPRESSION (Rumination)<br>RRS-BROODING (Rumination)<br>RRS-REFLECTION (Rumination)
Batmaz et al. (2014)	166 (60.2 %)	37.64 (12.27)	151 (63.6 %)	40.07 (12.6)	No comorbid anxiety	LESS-ACCEPT (Acceptance)<br>LESS-RUMINATION (Rumination)
Beblo et al. (2012)	39 (54 %)	38.9 (10.2)	41 (56 %)	37.7 (8.9)	Comorbid anxiety = 15.4 %	DERS-NONACCEPT (Acceptance)<br>EAQ-ACCEPT-NE (Acceptance)<br>EAQ-ACCEPT-PE (Acceptance)<br>ERQ-SUPPRESSION (Suppression)<br>EAQ-SUPPRESS-NE (Suppression)<br>EAQ-SUPPRESS-PE (Suppression)
Belleau et al. (2015)	16 (69 %)	33.19 (11.49)	16 (50 %)	31.13 (10.7)	No. of previous MDD episodes (M) = 3.06<br>Duration current MDD in months (M) = 21.63<br>Comorbid anxiety = 69 %	RRS-TOTAL (Rumination)
Brockmeyer et al. (2012)	41 (100 %)	35 (13)	60 (100 %)	34 (14)	-	DERS-NONACCEPT (Acceptance)<br>DERS-AWARE (Awareness)<br>DERS-CLARITY (Clarity)<br>DERS-GOALS (Tolerance)<br>DERS-IMPULSE (Tolerance)<br>DERS-STRATEGIES (Tolerance)<br>DERS-TOTAL (General ER abilities)

Appendix B: Table B.1 (current MDD) and B.2 (MDD in remission) with study characteristics

Brockmeyer et al. (2015) Trial I<br>Sample with episodic depression	30 (67 %)	38.57 (11.44)	30 (60 %)	38.33 (14.54)	Comorbid anxiety = 33.33 %	NAS-AVOIDANCE (Avoidance)<br>CBAS (Avoidance)
Brockmeyer et al. (2015) Trial II<br>Sample with chronic depression	30 (60 %)	39.8 (12.6)	30 (60 %)	38.33 (14.54)	Comorbid anxiety = 33.33 %	NAS-AVOIDANCE (Avoidance)<br>CBAS (Avoidance)
Chan et al. (2013)	25 (60 %)	40.96 (12.16)	25 (60 %)	38.88 (12.65)	-	RRS-TOTAL (Rumination)
Clark et al. (2011) Trial I	25 (48 %)	67.1 (7.5)	22 (36.36 %)	67.8 (5.1)	Comorbid anxiety = 64 %	SPSI-TOTAL (Problem solving)
Clark et al. (2011) Trial II	13 (38.46 %)	69.9 (7.5)	22 (36.36 %)	67.8 (5.1)	Comorbid anxiety = 46 %	SPSI-TOTAL (Problem solving)
Clark et al. (2011) Trial III	35 (62.86 %)	70.7 (8.4)	22 (36.36 %)	67.8 (5.1)	Comorbid anxiety = 49 %	SPSI-TOTAL (Problem solving)
Cooney et al. (2010)	14 (57.14 %)	40.6 (11.3)	14 (42.86 %)	34.6 (10.4)	No. of previous MDD episodes (M) = 6.5<br>Duration current MDD in months (M) = 14.3	RRS-TOTAL (Rumination)
Deveney & Deldin (2006)	19 (73.7 %)	32.18 (10.18)	18 (61.1 %)	33.8 (12.6)	Comorbid anxiety = 63.2 %	RRS-DEPRESSION (Rumination)<br>RRS-BROODING (Rumination)<br>RRS-REFLECTION (Rumination)
Dillon & Pizzagalli (2013)	12 (58.34 %)	31 (8.2)	24 (50 %)	34.42 (14.93)	No. of previous MDD episodes (M) = 2.33<br>Comorbid anxiety = 33.34 %<br>MDD onset age (M) = 18.58	RRS-DEPRESSION (Rumination)<br>RRS-BROODING (Rumination)<br>RRS-REFLECTION (Rumination)<br>ERQ-REAPPRAISAL (Reappraisal)<br>ERQ-SUPPRESSION (Suppression)
Dondaldson & Lam (2004)	36 (63.89 %)	45.6 (10.8)	36 (63.89 %)	43.2 (11.9)	No comorbid anxiety	RRS-TOTAL (Rumination)
Ehret et al. (2015)	30 (66.7 %)	41.03 (12.45)	30 (66.7 %)	39.17 (12.42)	No. of previous MDD episodes (M) = 1.47	ERSQ (General ER abilities)<br>FSCRS-REASSURE (Self compassion)<br>RSS (Rumination)
Farmer et al. (2003)	108 (65 %)	39.8 (1)	105 (74 %)	36.2 (1.2)	-	TCI-HA (Avoidance)
Fernando et al. (2014)	48 (54.17 %)	33.15 (8.89)	63 (65.1 %)	31.44 (9.98)	Comorbid anxiety = 31.25 %	DERS-TOTAL (General ER abilities)<br>ERQ-REAPPRAISAL (Reappraisal)<br>ERQ-SUPPRESSION (Suppression)

Appendix B: Table B.1 (current MDD) and B.2 (MDD in remission) with study characteristics

Fladung et al. (2010)	33 (72.7 %)	39.33 (10.3)	33 (72.7 %)	39.9 (10.04)	-	ERQ-REAPPRAISAL (Reappraisal)
Fletcher et al. (2013)	96 (59.1 %)	39.7 (11.4)	90 (59.1 %)	39.7 (11.4)	Comorbid anxiety = 20.8 %	CERQ-ACCEPTANCE (Acceptance)<br>CERQ-PLANNING (Problem solving)<br>BC-ACTIVECOPING (Problem solving)<br>BC-PLANNING (Problem solving)<br>CERQ-REAPPRAISAL (Reappraisal)<br>CERQ-PERSPECTIVE (Reappraisal)<br>BC-REFRAMING (Reappraisal)<br>CERQ-REFOCUS (Reappraisal)<br>RRS-TOTAL (Rumination)
Gibbs et al. (2009)<br>Trial I	18 (50 %)	67.9 (7.5)	19 (32 %)	69.2 (8.6)	Comorbid anxiety = 28 %	SPSI-PROBLEMOR (Problem solving)<br>SPSI-PROBLEMSOLV (Problem solving)<br>SPSI-AVOIDANCE (Avoidance)
Gibbs et al. (2009)<br>Trial II	27 (59 %)	74 (8.9)	19 (32 %)	69.2 (8.6)	Comorbid anxiety = 49 %	SPSI-PROBLEMOR (Problem solving)<br>SPSI-PROBLEMSOLV (Problem solving)<br>SPSI-AVOIDANCE (Avoidance)
Halvorsen et al. (2015)	37 (72.97 %)	37.49 (11.98)	50 (78 %)	38.06 (12.66)	-	RRS-TOTAL (Rumination)<br>TCQ-REAPPRAISAL (Reappraisal)
Halvorsen et al. (2009)	37 (72.97 %)	37.32 (11.93)	50 (80 %)	38.26 (12.64)	-	TCI-HA (Avoidance)
Hamilton et al. (2011)	17 (58.82 %)	45.06 (2.83)	17 (58.82 %)	41.94 (2.44)	No. of previous MDD episodes (M) = 2.88<br>Duration current MDD in months (M) = 51.18<br>Comorbid anxiety = 41.18	RRS-DEPRESSION (Rumination)<br>RRS-BROODING (Rumination)<br>RRS-REFLECTION (Rumination)

Hsu et al. (2007)	39 (100 %)	53.1 (6.4)	52 (100 %)	31.5 (5.9)	-	TPQ-HA (Avoidance)
Johnson et al. (2009)	20 (45 %)	21.9 (2.9)	24 (45.83 %)	20.6 (2.4)	-	RRS-BROODING (Rumination)<br>RRS-REFLECTION (Rumination)
Joorman et al. (2006)	64 (78.13 %)	35.78 (10.08)	91 (61.54 %)	35.46 (11.42)	-	RRS-BROODING (Rumination)<br>RRS-REFLECTION (Rumination)
Joorman & Gotlib (2008)	23 (69.57 %)	35.45 (10.83)	21 (66.67 %)	35.52 (12.49)	No comorbid anxiety	RRS-TOTAL (Rumination)<br>RRS-BROODING (Rumination)<br>RRS-REFLECTION (Rumination)
Joorman & Gotlib (2010)	22 (72.73 %)	36 (9.27)	32 (46.88 %)	38.85 (12.02)	No. of previous MDD episodes (M) = 5.79<br>Comorbid anxiety = 31.82 %	RRS-TOTAL (Rumination)<br>RRS-BROODING (Rumination)<br>RRS-REFLECTION (Rumination)<br>ERQ-REAPPRAISAL (Reappraisal)<br>ERQ-SUPPRESSION (Suppression)
Joorman et al. (2011)	26 (69.23 %)	46.73 (10.02)	27 (48.15 %)	38.42 (10.81)	-	RRS-TOTAL (Rumination)
Joorman et al. (2010) Trial I	23 (73.91 %)	39 (11.02)	24 (62.5 %)	34.96 (11.41)	Comorbid anxiety = 21.74 %	RRS-TOTAL (Rumination)
Joorman et al. (2010) Trial II	21 (57.14 %)	40.09 (9.67)	18 (72.22 %)	37.5 (9.37)	Comorbid anxiety = 42.86 %	RRS-TOTAL (Rumination)
Kircanski et al. (2015) Trial I	16 (100 %)	31.56 (10.28)	19 (100 %)	34.68 (9.88)	No cormorbid anxiety	RRS-BROODING (Rumination)
Kircanski et al. (2015) Trial II	20 (100 %)	35.50 (10.10)	19 (100 %)	34.68 (9.88)	Comorbid anxiety = 100 %	RRS-BROODING (Rumination)
Koch & Exner (2015)	36 (69.44 %)	47.05 (10.85)	36 (58.33 %)	32.81 (9.96)	Comorbid anxiety = 8.33 %	RRS-TOTAL (Rumination)
Lau et al. (2007)	43 (58 %)	39.2 (10)	36 (50 %)	34 (14.1)	Comorbid anxiety = 65.12 %	RRS-TOTAL (Rumination)
Lee et al. (2012) Trial I	30 (80 %)	44.93 (14.36)	314 (60.5 %)	42.54 (13.94)	No comorbid anxiety	TCI-HA (Avoidance)
Lee et al. (2012) Trial II	40 (67.5 %)	45.05 (10.72)	314 (60.5 %)	42.54 (13.94)	No comorbid anxiety	TCI-HA (Avoidance)
Levens et al. (2010)	24 (25 %)	41 (12)	24 (33.33 %)	37 (10)	Comorbid anxiety = 33.33 %	RRS-TOTAL (Rumination)<br>RRS-BROODING (Rumination)<br>RRS-REFLECTION (Rumination)

Mandell et al. (2014)	35 (60 %)	39.2 (12.3)	29 (68.97 %)	33.1 (10.1)	No. of previous MDD episodes (M) = 5.00	ECQ-REHEARSAL (Rumination)<br>MRQ (Rumination)<br>RNE-GENRUMIN (Rumination)<br>RNT-EMOT (Rumination)<br>RSS (Rumination)<br>RRS-TOTAL (Rumination)<br>RRS-BROODING (Rumination)<br>RRS-REFLECTION (Rumination)<br>SMRI-EMOT (Rumination)<br>SMRI-MOT (Rumination)<br>TCQ-REAPPRAISAL (Reappraisal)
Marchesi et al. (2008)	16 (100 %)	30.4 (3.2)	112 (100 %)	30.4 (4.7)	Duration current MDD in months (M) = 1.6	TAS-20 (Alexithymia)
Marchesi et al. (2014)	38 (50 %)	51.3 (11.7)	78 (80.8 %)	41.2 (11.8)	-	TAS-20 (Alexithymia)
Meiran et al. (2011)	9 (77.78 %)	44.4 (N.R)	9 (77.78 %)	41.1 (N.R)	-	RRS-TOTAL (Rumination)
Minaya & Fresán (2009)	38 (73.7 %)	34.3 (12.5)	89 (N.R)	N.R	Duration current MDD in months (M) = 42.33<br>No comorbid anxiety<br>MDD onset age (M) = 30.1	TCI-HA (Avoidance)
Minaya & Fresán (2009)	42 (71.4 %)	32.3 (9.2)	89 (N.R)	N.R	Duration current MDD in months (M) = 91.55<br>Comorbid anxiety = 100 %<br>MDD onset age (M) = 24.6	TCI-HA (Avoidance)
Nery et al. (2009)	45 (67 %)	37.9 (12.6)	60 (67 %)	38.4 (12.8)	No. of previous MDD episodes (M) = 8.1<br>Duration current MDD in months (M) = 40.8<br>Comorbid anxiety = 50.1 %	TCI-HA (Avoidance)
Nowakowska et al. (2005)	25 (68 %)	33.5 (12.3)	47 (61.7 %)	33.8 (14.2)	No comorbid anxiety	TCI-HA (Avoidance)

O'Kearney & Parry (2014)	24 (50 %)	39.6 (16.98)	24 (67 %)	35.8 (15.73)	-	IES-AVOIDANCE (Avoidance)
Ottaviani et al. (2015)	18 (66.67 %)	38.4 (12.1)	18 (61.11 %)	30.1 (10.5)	-	RRS-TOTAL (Rumination)
Ottenbreit et al. (2014)	60 (100 %)	42.05 (13.46)	30 (100 %)	36.53 (12.17)	Comorbid anxiety = 48.33 %	CBAS-TOTAL (Avoidance)
Pearson et al. (2010)	29 (65.52 %)	46.1 (13.8)	32 (68.75 %)	47.2 (17.3)	-	RRS-BROODING (Rumination)
					-	RRS-REFLECTION (Rumination)
Pu et al. (2012)	26 (57.69 %)	47.9 (19.2)	30 (60 %)	50.5 (19.7)	Duration current MDD in months (M) = 36.00 MDD onset age (M) = 36.8	CISS-T (Problem solving) CISS-A (Avoidance)
Regenbogen et al. (2015)	24 (N.R)	36.42 (12.01)	24 (N.R)	35.25 (9.8)	No comorbid anxiety	TAS-20 (Alexithymia)
Remmers et al. (2015)	29 (%)	37 (12)	27 (N.R)	44 (15)	-	RRS-BROODING (Rumination) RRS-REFLECTION (Rumination)
Riso et al. (2015)	27 (63 %)	39.1 (10.3)	24 (54.2 %)	40.7 (10.6)	-	RRS-TOTAL (Rumination)
Sasayama et al. (2011)	90 (50 %)	36.7 (10.2)	306 (50 %)	36.4 (11)	MDD onset age (M) = 29.3	TCI-HA (Avoidance)
Sigmon et al. (2006)	15 (80 %)	38.93 (13.2)	15 (80 %)	38.93 (13.2)	No. of previous MDD episodes (M) = 6.45	COPE-PROBLEMFOCUS (Problem solving) COPE-AVOIDANCE (Avoidance) COPE-ACCEPTANCE (Acceptance)
Sigmon et al. (2006)	15 (66.67 %)	38.93 (13.2)	15 (80 %)	38.93 (13.2)	No. of previous MDD episodes (M) = 7.52	COPE-PROBLEMFOCUS (Problem solving) COPE-AVOIDANCE (Avoidance) COPE-ACCEPTANCE (Acceptance)

Svaldi et al. (2012)	16 (100 %)	46.38 (7.29)	42 (100 %)	27.76 (6.34)	-	DERS-NONACCEPT (Acceptance)<br>DERS-AWARE (Awareness)<br>DERS-CLARITY (Clarity)<br>DERS-GOALS (Tolerance)<br>DERS-IMPULSE (Tolerance)<br>DERS-STRATEGIES (Tolerance)<br>DERS-TOTAL (General ER abilities)<br>ICARUS-ACCEPTFEEL (Acceptance)<br>ICARUS-ACCEPTSIT (Acceptance)<br>ICARUS-MINDFUL (Acceptance)<br>ERQ-REAPPRAISAL (Reappraisal)<br>ICARUS-SUPPRESS (Suppression)<br>ERQ-SUPPRESSION (Suppression)
Thompson et al. (2010)	39 (100 %)	27.7 (6.6)	41 (100 %)	27.2 (7)	-	COPE-PROBLEMSOLVING (Problem solving)<br>COPE-COGNITIVERESTR (Reappraisal)<br>RRS-TOTAL (Rumination)
Vorontsova et al. (2013)	30 (53.3 %)	42.5 (3.1)	30 (56.7 %)	40.4 (13.1)	-	AAQ (Avoidance)<br>RRS-TOTAL (Rumination)
Watkins & Baracaia (2002)	32 (75 %)	42.3 (12.8)	26 (61.54 %)	36.1 (12.2)	No. of previous MDD episodes (M) = 6.2<br>Duration current MDD in months (M) = 8.7<br>Comorbid anxiety = 28.1 %<br>MDD onset age (M) = 21.5	RRS-TOTAL (Rumination)

Appendix B: Table B.1 (current MDD) and B.2 (MDD in remission) with study characteristics

Watkins & Brown (2002)	14 (57.14 %)	42.9 (10)	14 (71.43 %)	36.2 (13.1)	No. of previous MDD episodes (M) = 5.2<br>Duration current MDD in months (M) = 17.2	RRS-TOTAL (Rumination)
Watkins & Moulds (2005)	32 (56.25 %)	41.8 (11.4)	32 (56.25 %)	39.8 (15)	No. of previous MDD episodes (M) = 6.4<br>Duration current MDD in months (M) = 16.2<br>Comorbid anxiety = 28 %<br>MDD onset age (M) = 21.8	RRS-TOTAL (Rumination)
Watkins & Moulds (2009)	52 (57.69 %)	40.69 (10.53)	49 (75.51 %)	36.55 (13.28)	No. of previous MDD episodes (M) = 6.32<br>Duration current MDD in months (M) = 21.0<br>Comorbid anxiety = 25 %<br>MDD onset age (M) = 22.08	RRS-TOTAL (Rumination)<br>RRS-BROODING (Rumination)<br>RRS-REFLECTION (Rumination)<br>TCQ-REAPPRAISAL (Reappraisal)<br>WBSI (Suppression)
Watson et al. (2013)	20 (70 %)	20.95 (4.42)	20 (75 %)	20.15 (2.23)	No. of previous MDD episodes (M) = 2.78<br>Duration current MDD in months (M) = 23.0<br>Comorbid anxiety = 55 %<br>MDD onset age (M) = 16.22	RRS-TOTAL (Rumination)
Zaninotto et al. (2015)	123 (69.9 %)	46.44 (13.52)	125 (51.2 %)	40.23 (16.47)	No. of previous MDD episodes (M) = 4.78<br>Duration current MDD in months (M) = 6.28<br>Comorbid anxiety = 49.6 %<br>MDD onset age (M) = 35.01	TCI-HA (Avoidance)

Zetsche et al. (2012)	20 (65 %)	45.7 (12.13)	25 (54 %)	36.6 (13.22)	Comorbid anxiety = 82 %	RRS-TOTAL (Rumination)<br>RRS-BROODING (Rumination)<br>RRS-REFLECTION (Rumination)

Note. RRS-DEPRESSION: Depression subscale of the Ruminative Response Scale; RRS-BROODING: Brooding subscale of the Ruminative Response Scale; RRS-REFLECTION; Reflection subscale of the Ruminative Response Scale; LESS-ACCEPT: Acceptance subscale of the Leahy Emotional Schema Scale; LESS-RUMINATION: Rumination subscale of the Leahy Emotional Schema Scale; DERS-NONACCEPT; Nonaccept subscale of the Difficulties in Emotion Regulation Scale; EAQ-ACCEPT-NE; Acceptance of negative emotions subscale of the Emotion Acceptance Questionnaire; EAQ-ACCEPT-PE: Acceptance of positive emotions subscale of the Emotion Acceptance Questionnaire; ERQ-SUPPRESSION: Suppression subscale of the Emotion Regulation Questionnaire; EAQ-SUPPRESS-NE: Suppression of negative emotions subscale of the Emotion Acceptance Questionnaire; EAQ-SUPPRESS-PE: Suppression of positive emotions subscale of the Emotion Acceptance Questionnaire; RRS-TOTAL: Total score of the Ruminative Response Scale; DERS-AWARE: Awareness subscale of the Difficulties in Emotion Regulation Scale; DERS-CLARITY: Clarity subscale of the Difficulties in Emotion Regulation Scale; DERS-GOALS: Goals subscale of the Difficulties in Emotion Regulation Scale; DERS-IMPULSE: Impulse control subscale of the Difficulties in Emotion Regulation Scale; DERS-STRATEGIES: Strategies subscale of the Difficulties in Emotion Regulation Scale; DERS-TOTAL: Total score of the Difficulties in Emotion Regulation Scale; NAS-AVOIDANCE: Avoidance subscale of the Need for Affect Scale; CBAS: Cognitive Behavioral Avoidance Scale; SPSI-TOTAL: Total score of the Social Problem Solving Inventory; ERQ-REAPPRAISAL; Reappraisal subscale of the Emotion Regulation Questionnaire; ERSQ: Emotion Regulation Skills Questionnaire; FSCRS-REASSURE: Reassure subscale of the Forms of Self-Criticizing/Attacking and Self-Reassuring Scale; RSS: Rumination on Sadness Scale; TCI-HA: Harm Avoidance subscale of the Temperament and Character Inventory; CERQ-ACCEPTANCE: Acceptance subscale of the Cognitive Emotion Regulation Questionnaire; CERQ-PLANNING: Planning subscale of the Cognitive Emotion Regulation Questoinnaire; BC-ACTIVECOPING: Active Coping subscale of the Brief COPE; BC-PLANNING: Planning subscale of the Brief COPE; CERQ-REAPPRAISAL: Reappraisal subscale of the Cognitive Emotion Regulation Questionnaire; CERQ-PERSPECTIVE: Perspective subscale of the Cognitive Emotion Regulation Questionnaire; BC-REFRAMING: Reframing subscale of the Brief COPE; CERQ-REFOCUS: Refocus subscale of the Cognitive Emotion Regulation Questionnaire; SPSI-PROBLEMOR: Problem orienting subscale of the Social Problem Solving Inventory; SPSI-PROBLEMSOLV: Problem solving subscale of the Social Problem Solving Intentory; SPSI-AVOIDANCE: Avoidance subscale of the Social Problem Solving Inventory; TCQ-REAPPRAISAL: Reappraisal subscale of the Thought Control Questionnaire; TPQ-HA: Harm Avoidance subscale of the Tridimentional Personality Questionnaire; ECQ-REHEARSAL: Rehearsal subscale of the Emotion Control Questionnaire; MRQ: Multidimensional Rumination Questionnaire; RNE-GENRUMIN: General Rumination subscale of the Rumination on a Negative Event questionnaire; RNT-EMOTION: Emotion-focused rumination subscale of the Rumination on Negative Thoughts; SMRI-EMOT: Emotionality subscale of the Scott Macintosh Rumination Inventory; SMRI-MOT: Motivation subscale of the Scott Macintosh Rumination Inventory; TAS-20: Toronto Alexithymia Scale; IES-AVOIDANCE: Avoidance subscale of the Impact of Event Scale; CBAS-TOTAL: Total score of the Cognitive Behavioral Avoidance Scale; CISS-T: Task Oriented subscale of the Coping Inventory for Stressful Situations; CISS-A: Avoidance subscale of the Coping Inventory for Stressful Situations; COPE-PROBLEMFOCUSED: The Problem Focused subscale of the COPE; COPE-AVOIDANCE: Avoidance subscale of the COPE; COPE-ACCEPTANCE: Acceptance subscale of the COPE; ICARUS-ACCEPTFEEL: Accept Feelings subscale of the Inventory of Cognitive Affect Regulation Strategies; ICARUS-ACCEPTSIT: Accept Situation subscale of the Inventory of Cognitive Affect Regulation Strategies; ICARUS-MINDFUL: Mindful Orientation of the Inventory of Cognitive Affect Regulation Strategies; ICARUS-SUPPRESS: Suppression subscale of the Inventory of Cognitive Affect Regulation Strategies;

Appendix B: Table B.1 (current MDD) and B.2 (MDD in remission) with study characteristics

COPE-PROBLEMSOLVING: Problem Solving subscale of the COPE; COPE-COGNITIVERESTR: Cognitive Restructuring subscale of the COPE; AAQ: Avoidance and Action Questionnaire; WBSI: White Bear Suppression Inventory; N.R: Information not reported.

Appendix B: Table B.1 (current MDD) and B.2 (MDD in remission) with study characteristics

Table B.2

Study characteristics - studies involving patients remitted from Major Depressive Disorder.

	MDD Sample		Healthy control sample			
Study (year)	N (% female)	M age (SD)	N (% female)	M age (SD)	Moderator(s)	Emotion regulation questionnaire(s)
Abravanel & Sinha (2015)	107 (68.2 %)	31.3 (9.49)	638 (56.1 %)	29.5 (9.24)	-	DERS-TOTAL<br>(General ER abilities)
Aker et al. (2014)	109 (100 %)	37.5 (11.3)	64 (100 %)	37.1 (12.3)	Comorbid anxiety = 23 %	ERQ-REAPPRAISAL (Reappraisal)<br>ERQ-SUPPRESSION (Suppression)<br>RRS-TOTAL (Rumination)<br>RRS-BROODING (Rumination)<br>RRS-REFLECTION (Rumination)
Benaseed et al. (2014)	47 (63.84 %)	34.47 (10.94)	120 (50 %)	32.04 (9.38)	-	TCI-HA (Avoidance)
Brockmeyer et al. (2012)	20 (80 %)	56 (12)	20 (75 %)	48 (17)	No. of previous MDD episodes (M) = 4.0	NAS-AVOIDANCE (Avoidance)
Ehret et al. (2015)	30 (66.7 %)	39.5 (12.13)	30 (66.7 %)	39.17 (12.42)	No. of previous MDD episodes (M) = 1.47	ERSQ (General ER abilities)<br>FSCRS-REASSURE<br>(Self compassion)<br>RSS (Rumination)
Ekinci et al. (2012)	80 (62.5 %)	36.8 (6.9)	80 (50 %)	31.6 (4.4)	No. of previous MDD episodes (M) = 3.3<br>Duration of remission in months (M) = 20.28<br>MDD onset age = 30.8	TCI-HA (Avoidance)

Appendix B: Table B.1 (current MDD) and B.2 (MDD in remission) with study characteristics

Farb et al. (2011)	16 (68.75 %)	44 (16)	16 (68.75 %)	39 (13)	No. of previous MDD episodes (M) = 4.6<br>Duration of remission in months (M) = 20.29<br>MDD onset age = 42.6	AAQ-R (Acceptance)<br>RRS-TOTAL (Rumination)
Halvorsen et al. (2015)	81 (87.65 %)	37.42 (9.61)	50 (78 %)	38.06 (12.66)	-	RRS-TOTAL (Rumination)<br>TCQ-REAPPRAISAL (Reappraisal)
Halvorsen et al. (2009)	53 (86.79 %)	36.79 (9.88)	50 (80 %)	38.26 (12.64)	-	TCI-HA (Avoidance)
Huffziger et al. (2013)	31 (70.97 %)	45.42 (7.98)	32 (68.75 %)	44.5 (7.86)	No. of previous MDD episodes (M) = 3.2<br>MDD onset age = 22.8	RRS-BROODING (Rumination)<br>RRS-REFLECTION (Rumination)
Joorman et al. (2006)	36 (72.22 %)	35.54 (8.48)	91 (61.54 %)	35.46 (11.42)	-	RRS-TOTAL (Rumination)
Joorman & Gotlib (2010)	47 (63.83 %)	35.88 (9.68)	32 (46.88 %)	38.85 (12.02)	No. of previous MDD episodes (M) = 3.62<br>No comoribid anxiety	RRS-TOTAL (Rumination)<br>RRS-BROODING (Rumination)<br>RRS-REFLECTION (Rumination)<br>ERQ-REAPPRAISAL (Reappraisal)<br>ERQ-SUPPRESSION (Suppression)
Nery et al. (2009)	15 (67 %)	40 (13.7)	60 (67 %)	38.4 (12.8)	No. of previous MDD episodes (M) = 3.9<br>Duration of remission in months (M) = 176.8<br>Comorbid anxiety = 26 %	TCI-HA (Avoidance)
Pearson et al. (2010)	42 (71.43 %)	44.4 (17)	32 (68.75 %)	47.2 (17.3)	-	RRS-BROODING (Rumination)<br>RRS-REFLECTION (Rumination)
Scherrer et al. (2014)	33 (78.79 %)	38.73 (12.01)	26 (76.92 %)	32.12 (10.52)	No. of previous MDD episodes (M) = 7.3<br>Duration of remission in months (M) = 168.0	RRS-TOTAL (Rumination)

Schiller et al. (2013)	19 (78.95 %)	23.6 (4.1)	19 (63.16 %)	27.9 (6.3)	No. of previous MDD episodes (M) = 1.6<br>Duration of remission in months (M) = 163.2<br>No comorbid anxiety	RRS-TOTAL (Rumination)
Ubl et al. (2015)	23 (69.6 %)	41.17 (12.08)	23 (60.9 %)	42.74 (12.19)	No. of previous MDD episodes (M) = 3.87<br>Duration of remission in months (M) = 121.68<br>Comorbid anxiety = 4.3<br>MDD onset age = 20.39	TPQ-HA (Avoidance)
Watkins & Baracaia (2002)	26 (69.23 %)	41.8 (9.9)	26 (61.54 %)	36.1 (12.2)	No. of previous MDD episodes (M) = 3.1<br>Comorbid anxiety = 11.5 %<br>MDD onset age = 27.6	RRS-TOTAL (Rumination)
Watkins & Moulds (2005)	33 (57.58 %)	31 (12.6)	32 (56.25 %)	39.8 (15)	No. of previous MDD episodes (M) = 4.6<br>Comorbid anxiety = 15 %<br>MDD onset age = 21.1	RRS-TOTAL (Rumination)
Watkins & Moulds (2009)	36 (69.44 %)	36.44 (12.67)	49 (75.51 %)	36.55 (13.28)	No. of previous MDD episodes (M) = 4.28<br>Comorbid anxiety = 16.67 %<br>MDD onset age = 23.58	RRS-TOTAL (Rumination)<br>RRS-BROODING (Rumination)<br>RRS-REFLECTION (Rumination)<br>TCQ-REAPPRAISAL (Reappraisal)<br>WBSI (Suppression)

Wolkenstein et al. (2014)	43 (72.09 %)	36.91 (13.35)	39 (58.97 %)	42.18 (13.27)	-	CERQ-RUMINATION (Rumination)<br>CERQ-REAPPRAISAL (Reappraisal)<br>CERQ-PERSPECTIVE (Reappraisal)<br>CERQ-REFOCUS (Reappraisal)<br>CERQ-ACCEPTANCE (Acceptance)<br>CERQ-PLANNING (Problem solving)
Zamoscik et al. (2014)	29 (68.97 %)	45.55 (7.45)	29 (72.41 %)	44.24 (8.09)	No. of previous MDD episodes (M) = 3.96<br>Comorbid anxiety = 6.9 %<br>MDD onset age = 23.14	RRS-BROODING (Rumination)<br>RRS-REFLECTION (Rumination)

Note. DERS-TOTAL: Total score of the Difficulties in Emotion Regulation Scale; ERQ-REAPPRAISAL; Reappraisal subscale of the Emotion Regulation Questionnaire; ERQ-SUPPRESSION: Suppression subscale of the Emotion Regulation Questionnaire; RRS-TOTAL: Total score of the Ruminative Response Scale; RRS-BROODING: Brooding subscale of the Ruminative Response Scale; RRS-REFLECTION; Reflection subscale of the Ruminative Response Scale; TCI-HA: Harm Avoidance subscale of the Temperament and Character Inventory; NAS-AVOIDANCE: Avoidance subscale of the Need for Affect Scale; ERSQ: Emotion Regulation Skills Questionnaire; FSCRS-REASSURE: Reassure subscale of the Forms of Self-Criticizing/Attacking and Self-Reassuring Scale; RSS: Rumination on Sadness Scale; AAQ-R: Acceptance and Action Questionnaire – Revised; TCQ-REAPPRAISAL: Reappraisal subscale of the Thought Control Questionnaire; TPQ-HA: Harm Avoidance subscale of the Tridimentional Personality Questionnaire; WBSI: White Bear Suppression Inventory; CERQ-RUMINATION: Rumination subscale of the Cognitive Emotion Regulation Questionnaire; CERQ-REAPPRAISAL: Reappraisal subscale of the Cognitive Emotion Regulation Questionnaire; CERQ-PERSPECTIVE: Perspective subscale of the Cognitive Emotion Regulation Questionnaire; CERQ-REFOCUS: Refocus subscale of the Cognitive Emotion Regulation Questionnaire; CERQ-ACCEPTANCE: Acceptance subscale of the Cognitive Emotion Regulation Questionnaire; CERQ-PLANNING: Planning subscale of the Cognitive Emotion Regulation Questionnaire.
